# WideEffHunter: An Algorithm to Predict Canonical and Non-Canonical Effectors in Fungi and Oomycetes

**DOI:** 10.3390/ijms232113567

**Published:** 2022-11-05

**Authors:** Karla Gisel Carreón-Anguiano, Jewel Nicole Anna Todd, Bartolomé Humberto Chi-Manzanero, Osvaldo Jhosimar Couoh-Dzul, Ignacio Islas-Flores, Blondy Canto-Canché

**Affiliations:** 1 Unidad de Biotecnología, Centro de Investigación Científica de Yucatán, A.C., Calle 43 No. 130 x 32 y 34, Colonia Chuburná de Hidalgo, Mérida C.P. 97205, Yucatán, Mexico; 2 Unidad de Bioquímica y Biología Molecular de Plantas, Centro de Investigación Científica de Yucatán, A.C., Calle 43 No. 130 x 32 y 34, Colonia Chuburná de Hidalgo, Mérida C.P. 97205, Yucatán, Mexico

**Keywords:** effectoromics, effector prediction, fungi and oomycetes, non-canonical effectors, effector-related domains, effector-related motifs

## Abstract

Newer effectorome prediction algorithms are considering effectors that may not comply with the canonical characteristics of small, secreted, cysteine-rich proteins. The use of effector-related motifs and domains is an emerging strategy for effector identification, but its use has been limited to individual species, whether oomycete or fungal, and certain domains and motifs have only been associated with one or the other. The use of these strategies is important for the identification of novel, non-canonical effectors (NCEs) which we have found to constitute approximately 90% of the effectoromes. We produced an algorithm in Bash called WideEffHunter that is founded on integrating three key characteristics: the presence of effector motifs, effector domains and homology to validated existing effectors. Interestingly, we found similar numbers of effectors with motifs and domains within two different taxonomic kingdoms: fungi and oomycetes, indicating that with respect to their effector content, the two organisms may be more similar than previously believed. WideEffHunter can identify the entire effectorome (non-canonical and canonical effectors) of oomycetes and fungi whether pathogenic or non-pathogenic, unifying effector prediction in these two kingdoms as well as the two different lifestyles. The elucidation of complete effectoromes is a crucial step towards advancing effectoromics and disease management in agriculture.

## 1. Introduction

Fungi and oomycete pathogens are the principal constraints to achieving world food security. These pathogens infect their hosts by releasing effectors, virulence-promoting molecules that manipulate a variety of host processes. Some effectors alter chromatin configuration, mimic host transcriptional activators, target host transcription factors, or interfere with the biosynthesis of phytoregulators, among other functions that alter host physiology. Effectors ultimately suppress plant defense responses, enabling the pathogen to form an association with the plant host which can result in disease.

Alternatively, effectors can have a positive impact on plant health when they are recognized by resistance receptors in the host. This recognition triggers the hypersensitive response which prevents further disease development. The current applications of effectors involve their use in genetic improvement programs [1,2], screening germplasm for effector cognates; primarily resistance proteins (R) [3] or susceptibility proteins that are targeted by effectors [4]. These efforts are propelling effectoromics as a key area of investigation in phytopathology.

Effector identification has been facilitated, in large part, by next generation sequencing and the accessibility of information deposited in public databases. Recently effectors have been identified from genomic, proteomic, and transcriptomic studies, particularly in pathosystems like that of *Pseudocercospora fijiensis—*banana [5], *Zymoseptoria tritici*—rice [6], *Ustilago hordei*—barley [7] and *Puccinia striiformis*—wheat [8] among others. Effector identification has become a staple of plant-pathogen investigations as the need heightens for novel and sustainable solutions to disease management. 

The identification of effector proteins has been based primarily on bioinformatic pipelines that use common or “canonical” criteria to facilitate effector identification. These canonical characteristics include the presence of a peptide signal, protein length ≤400 amino acids, cysteine-rich amino acid content (≥4 cysteines) and the absence of transmembrane domains (TMD) [9,10,11,12]. These criteria classify canonical effectors, the effector type predominantly identified in high-throughput effector studies of the last two decades. 

However, effector proteins that differ in one or more of these canonical criteria also exist and we will refer to them as “non-canonical effectors” (NCEs). Non-canonical effectors have been identified based on specific searches for motifs and domains that are associated with other characterized effectors [13,14,15], or because of overexpression data observed in transcriptomes of plant-pathogen interactions [8,16]. The effector Pi04314 (PexRD24) was identified while searching for the “RXLR” motif deduced from ESTs of the oomycete *Phytophthora infestans* during its interaction with potato and tomato. This non-canonical effector does not have a signal peptide in its sequence, but it has been shown to be secreted and then translocated to the host nucleus, promoting the host’s susceptibility to infection [17]. In the fungus, *Blumeria graminis*, a non-canonical effector called CSEP0064, found within a group of proteins containing a “RNase-like” domain denominated “RALPH”, has only two cysteines and was identified as part of a general search for domains within the small, secreted proteins of the fungus [18]. PsIsc1 and VdIsc1 are NCEs lacking signal peptides that were found by BLASTing sequences of known isochorismate synthases from other organisms and identifying their homologs in *Phytophthora sojae* and *Verticillium dahliae* [19]. Other NCEs surpass the 300 or 400 amino acid limit of canonical effectors. SAD1 of *Sporisorium reilianum* induces the loss of apical dominance in maize and *Arabidopsis* and is a NCE with 626 amino acids [20]. Similarly, the *Puccinia graminis* f. sp. *tritici* effector AvrSr35 is a secreted protein which interacts with the Sr35 cognate in wheat and is 578 amino acids in length [21]. AvrSr35 is not recognized as an effector by EffHunter or EffectorP 2.0. Like the other examples mentioned, these NCEs were proven to be effectors though functional characterization after identification. Other experimentally validated NCEs are not recognized by EffHunter or EffectorP 2.0 individually, or both [12]. The contribution of NCEs cannot be understated for the elucidation of complete pathogen effectoromes. 

Many recent reports continue to base their predictions of effectors on short amino acid lengths and cysteine richness [22,23], but others are searching by other means [8,13,14,15,16]. Available algorithms include the EffectorP machine learning (ML) series, among which the latest version, EffectorP 3.0, is able to classify effectors in the apoplast and cytoplast [24]. Sperschneider and Dodds (2022) [24], classified 176 true, experimentally-validated effectors; 64 were predicted apoplastic (extracellular) while a significantly larger 112 were predicted to be cytoplasmic, revealing a bias in effector identification based on canonical criteria. Another recent predictor, EffHunter, is a Perl script that is suitable for canonical effector classification since it strictly retrieves canonical effectors [12]. FunEffector-Pred, a ML algorithm, was trained with a similar number of proteins in both datasets to overcome the resulting bias of EffectorP which was trained with imbalanced positive and negative datasets [25]. Predector is another ML algorithm dedicated to fungal effectoromes, but for the predictive ranking of candidate effectors [26]. In the case of oomycete effectors, Nur et al. (2021) [27] constructed Effector-O, following a similar approach like that of FunEffector-Pred; this ML algorithm was trained with balanced 1:1 positive to negative training datasets, but Effector-O refines the prediction by retrieving the lineage-specific proteins. 

The identification of effectors can be challenging, but the advent of these algorithms has facilitated faster effector identification. All aforementioned algorithms were trained on validated true effectors, and these datasets comprise effectors that were identified following the criteria of canonical effectors. Previously, motifs such as RxLR-dEER and Y/F/WxC were once believed to be exclusive to oomycetes and were therefore excluded in the identification of fungal effectors. A turning point occurred when Godfrey et al. (2010) [28] found the motifs RxLR-dEER and Y/F/WxC within the N-terminal of 35 and 107 candidates, respectively, in *Blumeria graminis* f.sp. *hordei*. Recently, Zhang et al. (2020) [22] identified effectors in the transcriptome of the interaction of the basidiomycete fungus, *Puccinia triticina* and wheat. These authors used a Perl script that encompassed a motif search including RxLR found in oomycetes, [Y/F/W]xC found in powdery mildew, G[I/F/Y][A/L/S/T]R of flax rust, and [L/I]xAR, [R/K]CxxCx12H, and YxSL[R/K] of *Magnaporthe oryzae*, where they identified 635 effector candidates. Interestingly, part of them match the canonical criteria, but 45 had no cysteines at all, while 47 had only one. It is important to note that 244 cysteine-rich small, extracellular proteins of *P. triticina* had the [Y/F/W]xC motif, 24 had RxLR, 5 had G[I/F/Y][A/L/S/T]R, 64 had [L/I]xAR, and 2 had YxSL[R/K], indicating that these motifs are not exclusive to oomycetes. In contrast, Wood et al. (2020) [29] found effector candidates in the oomycete pathogen, *Bremia lactucae*, containing the WY domain but lacking the canonical RXLR motif. This shows that going beyond the canonical criteria allows for the expansion of effectoromes and the discovery of novel effectors. Likewise, Nur et al. (2021) [27], predicted 5814 candidates in the effectorome of *Phytophthora infestans*; they used a new identification approach which focused on seven biochemical characteristics of the N-terminus of the protein sequence instead of the classical oomycete effector motifs. The sum of the novel effectors found was one order of magnitude larger than the previously estimated effectorome of this pathogen. These results emphasize the need for an innovative algorithm that goes beyond classical effector identification, one that can identify both canonical and non-canonical effectors. Realistic estimations of pathogen effectoromes can provide a wide range of tools which can be exploited for disease control, for example, selecting non-redundant effector families, or designing strategies to target all members of a redundant family. 

We present a new effector identification tool called WideEffHunter. This is a user-friendly, modular and stand-alone algorithm for the identification of canonical and non-canonical fungal and oomycete protein effectors. The algorithm conducts a search in deduced proteomes for effectors containing domains or motifs, as well as proteins with homology to known fungal and oomycete effectors. Recent reports have shown in some fungal effectors the existence of previously believed oomycete effector exclusive motifs. Conversely, domains from fungal proteins have been identified in oomycete effectors [22,29,30]. Similarly, WideEffHunter found classical motifs of oomycete effectors in fungal effector candidates, meanwhile in *Phytophthora infestans*, the algorithm was able to identify LysM and other domains commonly found in fungal effectors. Characterization of effectoromes with EffHunter shows that the subset of canonical effectors comprises less than 10% of predicted effectoromes, suggesting that they represent just the tip of the iceberg in effectoromes. Interestingly, the comparison of the predicted effectoromes in fungi and oomycetes showed similar proportions of effectors containing domains, effectors containing motifs, and effectors that share homology with validated effectors, i.e., similar abundancies of effector conserved families. This suggests that evolution has shaped similar effectorome patterns in fungi and oomycetes, contrary to what is currently believed. It is worth mentioning that meanwhile other predictors were designed to be dedicated to one kingdom (fungi or oomycetes), or even to a particular lifestyle (for example only pathogens), the results for WideEffHunter support that this new predictor can be applied to both fungi and oomycetes, whether pathogenic or non-pathogenic to the plant host.

## 2. Results

### 2.1. Protein Databases

The true fungal effector dataset comprises validated effector proteins from diverse reports (Table 1); a non-redundant list of effectors was compiled which contains 228 true fungal effectors. The oomycete dataset was similarly constructed and it comprises 86 true oomycete effectors, as shown in Table 1. 

With respect to the non-canonical effectors, a comprehensive search of recent literature for novel, validated (true) non-canonical effectors was done. Thirteen NCEs were added to the fungal dataset, and three to the oomycete dataset. The lists of effectors comprising the fungal database are provided in Supplementary Table S1 while the list of oomycete effectors is provided in Supplementary Table S2.

### 2.2. In Silico Characterization of True Effectors

Effector identification is challenging, and even confusing at times, as different combinations of criteria can be used. The literature frequently states that not all effectors meet all the established effector criteria. Some predictions allow one or two TMDs, meanwhile others do not allow for proteins with any TMD. Similarly, the protein length cut-off used for effector identification is variable, between 200 to 400 amino acids. Other criteria such as cysteine content may also vary according to the study [5,12,32,33,34]. 

To help researchers prioritize the most important criteria for selecting or ranking effectors, as well as to identify properties that could aid in WideEffHunter’s design, true effectors were in silico characterized. 

Consistent with current criteria for effector identification, the majority (281 protein sequences, ~89%) was shorter than 400 amino acids, but 10.5% of them were not small proteins. The length of the largest known effectors is between 415 and 847 amino acids. Among them, KEX1, a yeast carboxypeptidase B-like killer toxin, has 847 amino acids. Other examples include PsCRN108, a CRN effector of *Phytophthora sojae*, which has 820 amino acids, and Jsi1, an effector of *Ustilago maydis* that interferes in host jasmonate/ethylene signaling and has a length of 641 amino acids. It is evident that large effectors occur both in fungal and oomycete kingdoms, but usually elude the current predictors. 

According to EffHunter, 142 proteins were canonical (45%), i.e., they had less than 400 amino acids, at least 4 Cys residues, a signal peptide for secretion and no TMD [12]. Non-canonical effectors (172 protein sequences, 54.7%) do not meet some of these criteria. Twenty-eight effectors had one or two TMDs (8.9%), meanwhile 3 effectors had 3–6 TMDs (Supplementary Tables S1 and S2). Only 11 effectors (3.5%) were predicted to have a Glycosylphosphatidylinositol (GPI) anchor domain. 

The order or ranking of the weight of each criterion based on the percentage of effectors that complied is as follows: No GPI (96.5%), no TMD (91.1%), sequence length less than 400 amino acids (89.4%), signal peptide (85%), extracellular (71.6%), ≥4% Cys (54.4%). Forty-five percent had only 0 to 3 Cys residues. Results are shown in Table 2.

To better evaluate the effectors of each of these kingdoms (fungi and oomycetes), the analyses were repeated on each database independently. Here, differences were evident between both groups. While 57% of fungal effectors were canonical, 86% of oomycete effectors were non-canonical (Table 3). With respect to fungi, only 7% of effectors had no cysteines, meanwhile 36% of oomycete effectors were cysteine-free. In total, 79.2% of oomycete effectors contained 3 cysteines or less, compared with 32.9% of fungal effectors. Conversely, 67% of fungal effectors had 4 cysteines or more, compared with 20.8% of oomycete effectors. Both classes coincide regarding TMDs, with the 90% of fungi and 93% of oomycete effectors having no TMD. Similarly, ~96 and 99% of fungi and oomycetes, respectively, had no GPI anchors (Table 3).

### 2.3. Functional Annotation of Fungal/Oomycete Effector Proteins: Domains and Motifs

Recently, with the intention of expanding effector prediction in fungal genomes, Huang et al. (2022) [13], Jaswal et al. (2021) [14] and Zhao et al. (2020) [15] conduced searches based on motifs, a strategy typically used to identify oomycete effectors (the motifs RXLR, ERR, LXL, FLAK, are usually associated with oomycete effectors). Conversely, motif- independent prediction of effectors was recently applied in oomycetes [27]. In both cases, the change of strategy rendered larger effectoromes.

To gain a better understanding of the role of domains and motifs in effector prediction, the fungal and oomycete effector databases were analyzed with the program InterProScan version 5.39–77.0 [35], which automatically and simultaneously searches in the databases of the modules CDD [36], PFAM [37], PRINTS [38], SMART [39] and TIGRFAM [40], among others; default parameter settings were used. 

Fifty-six domains were identified (Table 4). Some domains were identified only in fungal effectors (LysM, CFEM, cerato-platanin, among others), others in oomycetes (RXLR, Tetratricopeptide repeat domain, cystatin/monellin, RuvA domain), and others were shared among effectors of both kingdoms (glycosyl hydrolase, pectin lyase fold, NPP1, PROKAR lipoprotein, among others). The crinkler domain, usually associated with oomycete effectors, is present in RiNLE1, a nuclear-targeted effector of the arbuscular mycorrhizal fungus *Rhizophagus irregularis* [41]. This is a non-canonical fungal effector, since its length is 469 amino acids and no signal peptide is computationally deduced. The Localizer program predicts nuclear localization for RiNLE1, congruent with the report of Wang et al. (2021) [41]. Details of in silico characterization are provided in Supplementary Tables S1 and S2.

In total, 133 effectors contained at least one INTERPRO-domain; 49 domains were present in the fungal dataset (in 99 protein sequences), and 17 in the oomycete dataset (in 34 effectors). Details are included in Supplementary Tables S1 and S2. The most frequently occurring domains are related to carbohydrate binding or hydrolysis (LysM, glycosyl hydrolase, pectin lyase fold), since they play critical roles in host cell wall damage and pathogen cell wall-remodeling. Other effector functions are associated with entering the host cell, for example RXLR signatures in oomycete effectors, and fungal hydrophobins and cerato-platanins. In the important category of host defense suppression, the following domains were identified: crinkler, isochorismatase and chorismate mutase domain-containing effector. Various other domains are related to protein-protein interactions, which is expected since effectors need to bind their targets. Some effectors have domains characteristic of enzymes, such as lipases and different classes of proteases, meanwhile other effectors have protease-inhibitor domains.

Motifs have been used as probes to retrieve effector candidates, but usually only the most frequently occurring motifs are taken into consideration [13,14,15,22]. To date, no database of effector domains exists and the creation of this comprehensive list of effector domains represents a valuable tool for effectoromics. With respect to the number of known motifs, this list is still small. Further discovery of novel classes of effectors by genome mining and comparison of effectoromes may help to discover new effector-related domains.

In the positive dataset used here, no domains were identified in 181 effectors (57.6%): 129 from fungi (56.6%), and 52 (60.4%) from oomycete. All domain-free oomycete effectors belong to the non-canonical classification (Supplementary Table S2), but with respect to fungi, 64 non-canonical and 65 canonical effectors lacked domains. Table 5 shows a summary of these results, and details can be found in Supplementary Tables S1 and S2.

To test the regex designed here for domains, as well as the regex compiled from the literature regarding motifs, both regexes were used to mine the database of true effectors (positive dataset). As expected, these domains and motifs were found in the positive dataset (not shown). In fungi there were 110 hits, YFWxC being the most frequent (36), followed by motifs EAR (23), LysM (16), and [LI]xAR (16); curiously, 9 fungal true effectors had the RXLR motif. In the oomycete effectors, in addition to classical motifs for these microorganisms, the LysM domain was identified in 5 effectors and one was identified with a ToxA domain. 

To potentially find novel motifs, the sequences of the true effectors were analyzed using MEME suite. Table 6 shows the top 15 motifs found in fungal and oomycete effectors, respectively. The most frequent motif in fungi was MKFFTILL, found in 173 effectors (77.6% of fungal effectors; 55% considering the total database of 314 effectors). The other 14 motifs in fungal effectors were only present in 2 to 7 effectors. Regarding oomycetes, the most frequent motif was the RXLR motif found in 59 effectors (68.6%). The second most frequent was the motif MRLCYFLFVAAAAI, which was identified in 36 effectors, and the third, LYEHWHMRGCTPEHVYTILKLN, in 28 effectors. Similarly, the other 12 motifs were present in 2 to 7 effectors. For these most frequently occurring motifs (one for fungi and two for oomycete) found by MEME, a regex was created for them to be included in WideEffHunter. 

Analyses conducted here, even with these still limited sets of validated effectors, enable us to discover novel domains and motifs in fungal and oomycete effectors. Further discovery of novel classes of effectors through genome mining and effectorome comparative analysis may discover new effector-related domains and motifs.

### 2.4. Construction and Validation of WideEffHunter Algorithm 

The WideEffHunter code concatenates the mining of each regex for effector-related domains and motifs, including the three new motifs found here by MEME in the positive dataset (Table 6), and the results of Local Blastp against the database of true effectors. After pooling all hits, redundancy was eliminated which resulted in the predicted effectorome.

Table 7 shows validation results of WideEffHunter compared with SignalP 1.0 [9], SignalP 2.0 [31], SignalP 3.0 [24], and EffectorO [27], comparing predictions on the positive and negative datasets.

Since WideEffHunter includes the Blastp database of true effectors, it retrieves all sequences when tested on the positive dataset. On the contrary, tested on the negative dataset, WideEffHunter retrieves 1545 hits. This high number of “false positives” results in a very low F1 score. 

To improve the performance of WideEffHunter, analysis of the negative dataset using the MEME program was conducted. Supplementary Table S3 shows the top 15 motifs found which were used to refine the prediction of effectoromes. The number of hits from the positive dataset did not change because these motifs were not present in the dataset of known true effectors. Elimination of hits in the negative dataset containing these MEME motifs found in the negative sequence controls, reduced the number of false positives to 192. Specificity, precision, accuracy, false positive rate and F1 score parameters were all improved; these values were close to those shown by the three EffectorP versions (Table 7) and indicates that this version of WideEffHunter is sufficiently robust for effector prediction in fungal and oomycete proteomes.

**Figure 1:**
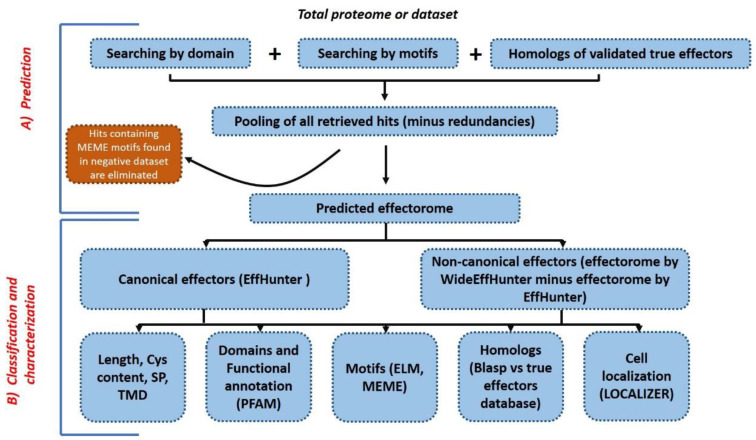
(**A**) Workflow to predict fungal and oomycete effectors with WideEffHunter. Positive database of true (validated) effectors comprises 228 fungal effectors and 86 oomycete effectors. Effector-related motifs were compiled from literature and enriched with motifs found in true effectors by the MEME program. (**B**) Classification and characterization of canonical and non-canonical effectors.

Figure 1 shows the WideEffHunter code and proposed downstream steps for effectorome characterization.

### 2.5. WideEffHunter Prediction of Effectoromes in Fungal and Oomycete Proteomes 

WideEffHunter was used to predict effectors on deduced proteomes of selected fungi and oomycetes. 

With respect to the oomycete effectoromes of *Bremia lactucae* and *Phytophthora infestans*, WideEffHunter predicted a similar number of effectors to that reported by Nur et al. (2021) [27] for *B. lactucae* (1812 vs. 1777 in the reference), and a lower number of effectors than that predicted by Nur et al. (2021) [27] for *P. infestans* (3811 in comparison with 5814 in the reference). In fungi, in all examples predicted here, WideEffHunter expanded the effectoromes: 3 times for *Puccinia triticina*, and 1.6 times for *Venturia inaequalis* (Table 8). In the case of the fungal endophytes *Pestalotiopsis fici* and *Xylona heveae*, and in the antagonist *Trichoderma harzianum*, the increases were significant, ranging from 6 to 18 times (Table 8). 

Curiously, the number of effector candidates in unfiltered WideEffHunter’s predictions is similar in most cases to predictions made by EffectorP 3.0, while the filtered predictions (that is, candidates without MEME motifs found in the negative dataset) in the pathogens *P. triticina*, *V. inaequalis*, *P. infestans* and *B. lactucae* were similar to those of EffectorP 2.0 (Table 8). Discrepancies between these two predictors were found with *T. harzianum*, *P. fici*, and *X. heveae*, in which WideEffHunter predicted larger effectoromes. Predictions of effectoromes of the non-pathogens *P. fici* and *X. heveae* by WideEffHunter were similar to EffectorP 1.0 predictions (Table 8).

Comparing the compositions of the effectoromes, we found that WideEffHunter shared ~60–70% hits with EffectorP 3.0 and EffectorO (Supplementary Table S4, tab “prediction”), but common hits were lower between WideEffHunter and EffectorP 3.0 for the non-pathogens (~40–46%). The lowest number of shared sets for WideEffHunter were observed in the effectoromes predicted by EffectorP 2.0 (~13–24%). Between 6 and 13% of effectoromes predicted by WideEffHunter were shared with those predicted by EffectorP 1.0, EffectorP 2.0, EffectorP 3.0, and EffectorO (Supplementary Table S4, tab “prediction”). 

Analysis of the catalogs of the effector candidates predicted by WideEffHunter revealed that >87% were non-canonical (Supplementary Table S4, tab “classification”). Around 80% lack TMDs and 64–80% are <400 amino acids in length, ~50% have at least 4 Cys residues, and less than 20% have signal peptides (Supplementary Table S4, tab “characterization”). The majority of effector candidates were predicted apoplastic (~50%), followed by nuclear (~30%), meanwhile proportions for mitochondria and chloroplast targeting were similar (~10–12%). Domains occurred in 40–60% of candidates and motifs were identified in 80–96%; the lesser contributing factor to the effectoromes was the subset of homologs of confirmed effectors (1.8–9.3%).

## 3. Discussion

Effectoromics is a central research area in plant pathology, but identification of effectors has been slow, difficult, and even confusing. There are several criteria used for effector identification, but not all effectors perfectly match the established criteria, making effector identification a challenge [9,30,34,43,44]. Effector identification pipelines are quite variable; the identification of effectors in fungi and oomycetes can permit the presence of one or two TMDs [33] or entirely exclude TMDs altogether [12,32]. They can have a protein size cutoff of 250 amino acids or less [5,33], 300 amino acids [43], or the upper limit can be set to 400 amino acids [12,25]. Some pipelines define effectors as having a cysteine content of ≥2% [45], ≥5% [46] while others consider at least 4 cysteine residues for effector candidature [12,23]. Recent pipelines were based on sequence homology within species of the same microbial genus [27,32], or the identification of domains or motifs, but the latter strategy has been exclusive to either fungi (domains) or oomycete (motifs) [29,47], but with no trans-kingdom application. Novel algorithms considering domains and motifs for both fungal and oomycete effectoromes prediction are necessary.

Fortunately, during recent years, the number of validated effectors has been increasing significantly. Sperschneider et al. (2018) [31] compiled 94 fungal and oomycete effector protein sequences in order to train EffectorP v2.0. More recently, Carreón-Anguiano et al. (2020) [12] compiled 150 effector sequences to validate EffHunter. In the present study we compiled 314 protein sequences taken from different datasets of true effectors: 228 from fungi, and 86 from oomycetes. This is the largest dataset of true effectors compiled to date. We found the absence of GPI anchors in 96.5% of effectors and the absence of TMDs in 90.7% of effectors. Additionally, sequence length was less than 400 amino acids in 89.4% of effectors, 85.1% had a signal peptide, 71.6% had extracellular localization, and 54.4% had a Cys content > 4% (Table 2). Cysteine content, one of the commonly used effector identification criteria, is not met by almost 50% of the true effectors. Both fungi and oomycete coincide in that >90% of effectors lack TMDs and no GPI anchors. This knowledge about the weight of each criterion will help researchers make better decisions when they are selecting effector candidates or creating new algorithms.

According to our analysis using WideEffHunter, around 50% of known fungal effectors are canonical, while in oomycetes, more than 85% are non-canonical. These differences may be attributed, in part, to genuine evolutionary differences among effectors in these kingdoms; for example, while most known fungal effectors are secreted to the apoplast, the majority of described oomycete effectors are translocated into the host cell [48]. However, the observed differences may result from a bias in the pipelines used until this point for the identification of effectors in these kingdoms; in fungi, effectors are usually identified based on protein length and cysteine content, while in oomycetes, the search is usually based on motifs such as RXLR, ERR, LXL, and FLAK [22,25,48]. 

During the characterization of validated effectors (positive datasets), we compiled a comprehensive list of motifs and domains present. It is important to mention that no databases of effector domains existed before. In previous studies, the predictions only considered a few domains such as LysM or CFEM, by mining proteomes with regular expressions or Hidden Markov Models [13,14,15,49,50]. The newly created database of effector-related domains, together with the motif database compiled from literature, represent valuable tools for effectoromics. The characterization of true effectors facilitated the identification of new effector features, such as the motif MKFFTILL which was present in 173 fungal effectors, and RHLRSHYQDEE, present in 59 oomycete effectors. The potential importance of novel effector motifs, especially in fungi, may be evidenced by citing the comments of He et al. (2020) [48]; in their words “a breakthrough for oomycete pathogens was the identification of the conserved amino acid motifs RxLR and LFLAK. These motifs define sets of several hundred intracellular effectors and have led to an upsurge in research on effector–host target interactions. For fungal plant pathogens, there are no such universal motifs, so the identification of *bona fide* intracellular effectors is a labor-intensive process initiated by the broader bioinformatic prediction of secreted proteins”. Therefore, these motif sequences enrich the current pool of computational tools available for effector identification. 

As mentioned before, domains and/or motifs have recently been used as probes to retrieve effector candidates such as the frequently occurring LysM and CFEM domains (fungi), and RXLR, LFLAK, Y/F/WxC, and CRN motifs (oomycetes). However, to date, only a few studies have employed this new “out-of-box” strategy, where motifs were the motor for fungal effector identification [13,14,15], or, in contrast, motif-independent searches for oomycete effectors were executed [27]. This strategy identified 719 RXLR-like, 19 CRN-like, and 138 Y/F/WxC new effector candidates in the fungus, *P. graminis*, in addition to the previously predicted effectorome following classical fungal effector identification methods [15]. This suggests that these classes of effectors are not exclusive to oomycetes and may contribute greatly to fungal effectoromics. These strategies have not only helped identify novel effectors, but have sometimes increased the number of known effectors by one order of magnitude, as was the case for *P. infestans* with an initial 563 effectors [51] which was further increased to 5814 [27]. According to WideEffHunter, fungal effectoromes comprise ~90% motif-containing effectors (similar to the proportion found during our analysis in oomycetes), and oomycete effectoromes comprise ~47–49% domain-containing effectors (similar to the proportion found here in fungi); likewise, the proportion of nuclear-targeted effector candidates are not very different between fungi and oomycetes. Actually, it is noteworthy that the percentages of effectors for each particular characteristic are similar among the predicted effectoromes (Supplementary Table S4, tabs “classification” and “characterization”), which suggests that contrary to current belief, the effectoromes in fungi and oomycetes have followed similar evolutionary histories. The occurrence of shared motifs and domains can facilitate the development of bioinformatics tools suitable for both kingdoms and will enable us to clarify whether fungi and oomycete effectoromes follow different evolutive histories, or the differences resulted from biases in previous identification methods.

Omics studies, especially transcriptomics and proteomics of plant-pathogen interactions, have largely contributed to the discovery of novel, non-canonical effectors (Table 2 and Table 3), but these effectors are still the most elusive for computational identification. WideEffHunter was constructed to expand effectoromes, combining domains and motifs found either in fungal or oomycete effectors for the identification of both canonical and non-canonical effectors. The in silico characterization of 172 NCEs (98 from fungi and 74 from oomycetes), shows that 56 have functional domains but 116 effectors do not (Table 5). In agreement with this result, recently in *Fusarium sacchari*, 41% of predicted effectors had no known domains or motifs [13]. In order to widen the prediction capacity of WideEffHunter, the database of known true effectors was nested in WideEffHunter as a search tool, added to the regex for motifs and domains. 

Validation of WideEffHunter was carried out in two runs. In the first, it retrieved 1545 hits from the negative dataset (“false positives”) and had poor performance parameters (F1 score 0.287). After the elimination of hits that contained motifs found by the MEME program in the negative dataset, the retrieved hits from the negative control decreased to 192. All parameters of WideEffHunter were improved with that step (Table 7) and attained parameter values closer to those shown by the EffectorP predictors. It was observed that EffectorO retrieved 781 hits from the negative dataset. We checked the composition of the retrieved hits from the negative dataset by WideEffHunter and EffectorO and observed that most of them contain the motifs RXLR, EAR and CRN in the expected N-terminal position on the effector proteins. Additionally, WideEffHunter hits were comprised of 52 false positives with LysM domains (not shown). It is worth mentioning that the EffectorO ML algorithm was created for mining oomycete proteomes, and the overestimation observed here was because we analyzed the uploaded proteomes in Fasta files online with default settings but did not later select those candidates with lineage-specific phylogenetic distribution. That tool may improve EffectorO prediction, but we decided not to include it since the EffectorO script discards all hits that match with homologs in fungi and we would therefore not be able to apply this to fungal proteomes. 

The possibility exists that some proteins in the negative dataset used in the present study are undiscovered effectors, since this set contains proteases, lipases, scytalone dehydratases, among others. Construction of negative datasets is really challenging since many non-effectors could be undiscovered effectors. Recently, in training the ML algorithms Predector and EffectorP 2.0, the authors included proteins from saprophytes and symbionts in the negative datasets, but the number of reports showing the presence of effectors in saprophytes and symbionts is currently increasing [52,53], and these predictors are most likely ruling out many potential true effectors. However, authors of EffectorP algorithms acknowledged that EffectorP 2.0 was improved in pathogen effector identification, since it excluded many proteins that are shared with non-pathogens compared to EffectorP 1.0 [31]. In congruence with what was expected, EffectorP 2.0 predicted lower effectoromes than WideEffHunter for the antagonist *T. harzianum*, and the endophytes *P. fici* and *X. heveae*. WideEffHunter also expanded effectoromes in comparison with Queiroz and Santana (2020) [43], since these authors restricted the identification to small, secreted cysteine-rich proteins with no conserved domains, containing a nuclear localization signal and repetitive sequences. 

Curiously, predictions of WideEffHunter for pathogenic fungi and oomycete is closest to predictions made by EffectorP 2.0, meanwhile WideEffHunter predictions for endophytes match with predictions of EffectorP 1.0. This is congruent with the fact that EffectorP 1.0 was not designed to filter saprophytes. Therefore, it seems that WideEffHunter is suitable for both pathogenic and non-pathogenic fungi and oomycetes. We also observed that, on various proteomes, the prefiltered results of WideEffHunter are close to the results of EffectorP 3.0. 

As an additional test to evaluate its performance, WideEffHunter was used to predict effectoromes that were previously predicted following different criteria, and WideEffHunter performed well in these predictions (Table 8). This reinforces that while other predictors are specialized for use in one kingdom, or even for a particular lifestyle (e.g., pathogens), WideEffHunter suitably works on different lifestyles in fungal and oomycete kingdoms. Around 60% of effector candidates predicted by WideEffHunter are shared with those predicted by EffectorP 3.0 or EffectorO (Supplementary Table S4). Therefore, WideEffHunter retrieves ~30–40% of novel candidates, expanding effectoromes. Effectors are so variable that no predictor can detect all potential candidates so authors usually recommend combining predictors [12,26,27,31]. Fungi and oomycetes are filamentous species that share similarities, but also differ from each other [48,54,55] so the prediction of their effectoromes has also followed different routes [25,27]. The WideEffHunter algorithm unifies the prediction of fungal and oomycete effectors.

Classification of effector candidates predicted by WideEffHunter shows that canonical effectors comprise less than 10% of effectoromes, suggesting that NCEs play a more important role than we previously believed. 

Some effectors have been reported as elusive for current predictors; for example, PIIN 08944, and AvrSr355 which are not recognized by EffHunter or EffectorP 2.0; SAD1 and BEC1054, that are not recognized by EffHunter, and Mg3LysM, BEC1019 and CSEP0105, that are not recognized by EffectorP 2.0. WideEffHunter was able to retrieve all of these effectors since one of the retrieving tools is homology-based Blastp against the true effectors database. Effector candidates with homology represent 1.8 to 9% of effectoromes (Supplementary Table S4, tab “characterization”), indicating that this additional tool improved the performance of WideEffHunter. This result is congruent with the limited number of conserved families known currently in effectoromics. Some effectors that are widely distributed in fungi are Avr4, Ecp2, Ecp6, and NIS1, among others [30]. In oomycetes, the HaRxL23 [56], RXLR effectors [57], as well as CRN12_997 and other CRN effectors are conserved [58]. As more is revealed about complete effectoromes, more conserved families of effectors will be revealed.

Since effectoromics is continuously expanding, WideEffHunter was constructed modularly (Figure 1), giving researchers the opportunity to use the WideEffHunter algorithm as it was constructed, or to eliminate a particular regex of any domains or motifs for genome mining in their organism of choice. The list of motifs, domains and validated effectors are still limited, but further comparison of effectoromes may reveal new effectors, domains and motifs. The WideEffHunter algorithm also allows users to continuously feed it with new data, keeping the algorithm updated and making WideEffHunter a tool that continuously catalyzes the discovery of novel effectors.

## 4. Materials and Methods

### 4.1. Data Protein Collection

The dataset of true fungal and oomycete effectors was constructed by combining diverse datasets of experimentally validated effectors compiled in Carreón-Anguiano et al., (2020) [12], Jones et al., (2021) [26], Nur et al., (2021) [27], Sperschneider et al., (2018) [31], Wang et al., (2020) [25]. Additionally, 18 validated effector proteins were taken directly from their individual reports (sequences are provided in Supplementary Tables S1 and S2).

For the conversion of fasta files to text files and/or vice versa, the “Seqret” tool in the European EMBOSS platform (https://www.ebi.ac.uk/Tools/sfc/embossseqret/) was used. For the generation of a database in tabular format, the sequences in the fasta file were converted using a Python v2.7.18 script, separating the header and sequence motif information in a tab delimitated format.

### 4.2. In Silico Characterization of Effectors

A comprehensive analysis of each of the following effector criteria was done for the 228 fungal and 86 oomycete effectors belonging to the positive datasets: number of amino acids (length), cysteine residue number and percentage were analyzed with ProtParam tool at Expasy (https://web.expasy.org/protparam/; access 20 January 2022), transmembrane domain prediction with TMHMM [59], and the presence of signal peptides with SignalP 5.0 [60]. Protein subcellular localization was analyzed using LOCALIZER [61], and cell wall-bounded proteins were identified with PredGPI [62]. All programs were run with default parameters. 

Canonical effectors were identified with the EffHunter algorithm [12] and the remaining proteins, (WideEffHunter prediction minus EffHunter prediction), were classified as non-canonical. 

For functional domain identification, effector sequences were analyzed with PFAM [37] and InterPro [63]. Motifs were identified using MEME suite [64] and were manually searched for using motifs described in previous literature [9,10,13,15,65,66]. Functional annotation was carried out using the PFAM module in InterproScan STANDALONE mode [37].

### 4.3. Construction of Databases 

Three databases were constructed: one for effector-related domains, another for effector-related motifs, and the third for the true validated effectors. 

#### 4.3.1. Database of Domains

Consensus sequences of the domains (for example LysM, CFEM, etc.) were downloaded from the “Simple Modular Architecture Research Tool” (SMART) web platform [39], selecting the consensus sequences with a value of 80%. Using “search SMART”, the information pertaining to the domains and the alignment consensus sequences were obtained. Consensus alignment sequences downloaded from SMART (Regex) were translated to regular expressions (regex) in Perl language (Supplementary Tables S5.1 and S5.2).

#### 4.3.2. Database of Motifs

Regexes for effector-related motifs were taken from Huang et al. (2022) [13], Zhao et al. (2020) [15], Liu et al. (2019) [66], Sonah et al. (2016) [10], Adhikari et al. (2013) [65] and Sperschneider et al. (2016) [9]. In addition to these motifs obtained from the literature, three novel motifs identified by MEME were included: the MKFFTILL, motif found in fungi, and two oomycete motifs, MRLCYFLFVAAAAI and LYEHWHMRGCTPEHVYTILKLN. Regexes of motifs were designed in Perl language.

The databases of domain and motifs were created in tabular format as stated above. 

#### 4.3.3. Database of True Effectors

The list of amino acid sequences of fungal and oomycete validated effectors were converted to Fasta Format, and later converted to an indexed database using the following Linux command for BLAST “*$:formatdb -i <Fasta.fasta> -p T –o T*”.

### 4.4. Construction of WideEffHunter

WideEffHunter algorithm was constructed in Bash language 5.0.17 concatenating the different regexes (in Perl 5.30.0) corresponding to effector-related domains and motifs; input and output files are in Fasta format. Effector hits retrieved from the search for domains were pooled with the hits retrieved by the other criterion, the presence of motifs). The third search was performed using Local Blastp against the database of true effectors, and the hits were also pooled with the list of effector candidates retrieved in the domains and motifs searches. Redundancies were eliminated with the command pipeline “*$: cat <File.txt> | sort | uniq*”. The resulting list was considered to be the predicted effectorome of the fungus or oomycete under study. 

All databases in FASTA and TAB format, positive protein datasets, open-source codes and accessory scripts can be found on the GitHub platform (https://github.com/Gisel-Carreon) and on the home page of Dr. Blondy Canto Canché (https://www.cicy.mx/unidad-de-biotecnologia/investigador/blondy-beatriz-canto-canche).

The command to execute WideEffHunter once it is installed in a linux/unix system, is “*$: ./WideEffHunter.sh*”.

It is worth mentioning that each step is modular; therefore, users can use the entire WideEffHunter as it was originally constructed for automatic prediction, or the user can delete a particular regex or database; likewise, users can add a regex for new effector-related domains and motifs, as well as upload newly discovered effectors to the positive dataset. In this way, WideEffHunter can be regularly updated.

### 4.5. Validation of WideEffHunter

For the validation of WideEffHunter, the positive dataset was used containing a total of 314 true effectors; 228 from fungi and 86 from oomycetes. 

For the negative control, the dataset used in Carreón-Anguiano et al. (2020) [12] was used. This dataset contains 4528 protein sequences of different lengths, presence/absence of signal peptide and TMD. We selected this negative dataset because it was not constructed selecting proteins from saprophytes, as in other reports [26,31]. Saprophytes also contain effectors [52,53], and negative datasets containing their proteins to train algorithms may rule out novel, true effectors. Furthermore, during the validation of algorithms like WideEffHunter, it may result in higher numbers of “supposedly false positives”. 

Motifs in proteins in the negative dataset were found through analysis with MEME; “negative exclusive” motifs were identified by searching for these motifs in the database of true effectors. To refine the prediction of false positives by WideEffHunter, the hits retrieved with the pipeline “domains + motifs + homologs of true effectors” were filtered eliminating those containing MEME motifs exclusive to negative control proteins. 

The numbers of true positives, true negatives, false positives, and false negatives, were used to calculate sensitivity, specificity, precision and accuracy parameters as well as the F1 score, a parameter widely used to measure and compare performances of different software/pipelines [12,31]. 

The performance of WideEffHunter was compared with that of EffectorP 1.0 [9], EffectorP 2.0 [31], EffectorP 3.0 [24] and EffectorO [27].

### 4.6. Prediction of Effector Proteins in Fungal and Oomycete Genomes

For comparative analysis, recent reports that predict effectors using domains and motifs were selected. The genomes (rather deduced proteomes) that were searched with WideEffHunter were from the oomycetes *P. infestans* and *B. lactucae* [27], and the fungal pathogens *P. triticina* [15] and *V. inaequalis* [42]. In addition, the fungal endophytes *P. fici* and *X. heveae* [43], and the antagonist *T. harzianum* [12], were included.

Subsequently, effector candidates were classified as canonical or non-canonical using EffHunter. The number of non-canonical effectors was estimated by subtracting the prediction by EffHunter from the prediction by WideEffHunter. 

Both classes, canonical and non-canonical effector candidates, were further in silico characterized in terms of: (a) number of amino acids, cysteine content, signal peptide, TMDs; (b) identification of effector-related domains; (c) identification of effector-related motifs and potential function (annotation); (d) homologs of true effectors; (e) cell localization.

## 5. Conclusions

WideEffHunter, an algorithm that predicts effectors based on effector-related domains and motifs, as well as homology to known validated effectors, is suitable for the retrieval of whole effectoromes (canonicals and non-canonical effector candidates) in pathogenic and non-pathogenic fungi and oomycetes. This is a user-friendly and modular algorithm that can be updated continuously with new domains, motifs and novel effectors, providing a powerful tool to strengthen effectoromics research.

## 6. Patents

The present algorithm was certified at Mexican Public Copyright Registry with the registration number 03-2022-101112004700-01.

## Figures and Tables

**Table 1:** List of positive datasets compiled in the present study.

Type of Dataset	Sequence Origin	Protein Sequences	Reference
Fungal	EffHunter	134	[12]
	EffectorP v2.0	20	[31]
	FunEffector-Pred	25	[25]
	Predector	36	[26]
	- *	13	This study
Oomycete	EffHunter	9	[12]
	EffectorO	74	[27]
	- *	3	This study

* Sequences obtained from this study.

**Table 2:** Summary of in silico characterization of canonical and non-canonical fungal/oomycete true effectors.

	Canonical	Non-Canonical	Total	Percentage (%) *
Length <400 amino acids	142	139	281	89.5
Length >400 amino acids	-	33 **	33	10.5
zero cysteine	-	47	47	15
1–3 cysteines	-	96	96	30.8
4–8 cysteines	111	19	130	41
9–10 cysteines	15	0	15	4.7
11–16 cysteines	14	7	21	6.8
17–19 cysteines	0	1	1	0.3
20–25 cysteines	2	2	4	1.3
No signal peptide	-	47	47	15
Signal peptide	142	125	267	85
No TMD	142	143	285	90.7
TMD	-	29 ^&^	29	9.3
No GPI	133	170	303	96.
GPI-anchor	9	2	11	3.5
Extracellular	113	112	225	71.6
Intracellular	29	60 ^#^	89	28.4

* Considering 314 effectors as the total (142 canonical and 172 non-canonical). ** Rank between 415 and 847 amino acids. ^&^ 1–6 TMDs. ^#^ cytoplasmic or organelle localized.

**Table 3:** Characterization and comparison of fungal and oomycete effectors.

	Fungal	% in Fungal Database *	Oomycete	% in Oomycete Database **	Total	% in Fungal + Oomycete Database
Canonical	130	57	12	13.9	142	45.2
Non-canonical	98	43	74	86.1	172	54.8
Length <400 amino acids	211	92.5	70	81.4	281	89.4
Length >400 amino acids	17	7.5	16	18.6	36	10.6
zero cysteine	16	7	31	36.1	47	15
1–3 cysteines	59	25.9	37	43.1	96	30.6
4–8 cysteines	116	50.9	14	16.4	130	41.4
9–10 cysteines	14	6.1	1	1.1	15	4.7
11–16 cysteines	19	8.4	2	2.2	21	6.7
17–19 cysteines	0	-	1	1.1	1	0.3
20–25 cysteines	4	1.7	0	-	4	1.3
No signal peptide	17	7.5	30	34.9	47	14.9
Signal peptide	211	92.5	56	65.1	267	85.1
No TMD	205	89.9	80	93	285	90.7
TMD	23	10.1	6	7	29	9.3
No GPI	218	95.6	85	98.8	303	96.5
GPI-anchor	10	4.4	1	1.2	11	3.5
Extracellular	174	76.3	51	59.3	225	71.6
Intracellular	54	23.7	35	40.7	89	28.4

* Total was 228 protein sequences; ** total was 86 protein sequences.

**Table 4:** Functional domains identified in fungal and oomycete effectors.

Domain	Fungi	Oomycete	Total	Function
Glycosyl hydrolase	13	2	15	Glycoside hydrolase
LysM	13	-	13	Peptidoglycan binding
RXLR signature	-	11	11	Effector translocation into host cells
Pectin lyase fold	7	1	8	Pectolytic enzyme, pectin lyase, which acts as a virulence factor.
RlpA	7	-	7	Transglycolase, endoglucanase. Lytic transglycosylase with a strong preference for naked glycan strands
CFEM domain	6	-	6	Fungal specific cysteine-rich domain, found in some proteins involved in fungal pathogenesis
NPP1	4	1	5	Necrosis-inducing protein
Cerato-platanin	4	-	4	Functional similarities with expansins; may facilitate the mechanical penetration of fungi
Peptidase_A1	4	-	4	Protease
Metalloprotease	4	-	4	Protease
Crinkler	1	3	4	CRN proteins participate in processes controlling plant cell death and immunity
PROKAR lipoprotein	1	3	4	Relatedto prokaryotic membrane lipoproteins. Domain present in enzymes, inhibitors, transporters, structural proteins, and virulence factors
Chitin binding Peritrophin-A domain	3	-	3	A six-conserved-cysteine domain found in chitin binding proteins, chitinases
Elicitin signature	-	3	3	Signature present in some oomycete extracellular avirulence or virulence factors
Nudix Box	1	1	2	Present in pyrophosphohydrolases, isopentenyl diphosphate isomerases, adenine/guanine mismatch-specific adenine glycosylases (A/G-specific adenine glycosylases), and non-enzymatic activities involved in protein/protein interaction and transcriptional regulation
Fungal cellulose binding domain	2	-	2	Cellulose binding
Aspartic peptidase, active sit	2	-	2	Protease
Thiamine binding	2	-	2	Role in protein-protein interactions
alpha/beta hydrolase	2	-	2	Domain in hydrolytic enzymes of widely differing phylogenetic origin and catalytic function
Egh16	2	-	2	Virulence factor
Nis1	2	-	2	Play critical roles in plant-microbe interactions (be required for pathogen virulence), but specific functions are still unknown
Fungal hydrophobin signature	2	-	2	Spontaneously assemble into amphipathic layers at hydrophilic-hydrophobic interfaces
ToxA	2	-	2	Proteinaceous host-selective toxin. Cause cell death in susceptible wheat cultivars
Subtilisin	2	-	2	Peptidase S8
Chymotrypsin	-	2	2	Peptidase S1A, serine protease
Kazal	-	2	2	Serine protease inhibitor
Concanavalin A-like lectin	1	1	2	Carbohydrate binding
Cutinase signature	1	1	2	Cutin alpha/beta hydrolase
Domain of unknown function	1	1	2	No characterized function
Zinc finger CCHC-type	1	1	2	High-affinity binding to single-stranded nucleic acids, especially single-stranded RNAs.
RAB5, RABX5	1	-	1	Key factor in early endocytosis
Hce2	1	-	1	Putative necrosis-inducing factor
M35_deuterolysin_like	1	-	1	Lysine-specific metallo-endopeptidase
Alternaria alternata allergen 1	1	-	1	In fungal exclusive protein family, with unknown function. Commonly secreted by fungi in *Alternaria* genus
ToxB	1	-	1	Proteinaceous host-selective toxin that causes chlorophyll degradation and foliar chlorosis
Isochorismatase	1	-	1	Conversion of isochorismate into 2,3-dihydroxybenzoate and pyruvate; disrupts the plant salicylate metabolism pathway
Fungal_RNase	1	-	1	Guanine-specific ribonuclease
VPS9	1	-	1	Vacuolar protein sorting-associated protein
Beta-lactamase-inhibitor protein II	1	-	1	Inhibitors of class A β-lactamases
Allergen V5/Tpx-1 family signature	1	-	1	Domain present in mammalian testis-specific protein (Tpx-1); venom allergen 5 from vespid wasps and venom allergen 3 from fire ants. The function in pathogen proteins is unclear
Rhomboid domain	1	-	1	Conserved domain in some proteases, that cleaves type-1 transmembrane domains using a catalytic dyad composed of serine and histidine. Peptidase S54
Mitochondrial carrier domain	1	-	1	Mitochondrial basic amino acids transporter
Integrin	1	-	1	Ubiquitously cell surface receptors involved in regulating the cell interaction
AroQ	1	-	1	Chorismate mutase. Suppression of plant immunity by manipulating the salicylic acid pathway
Pyridoxal phosphate-dependent transferase, major domain	1	-	1	Cys/Met metabolism
PAN domain	1	-	1	Mediation of protein-protein and protein-carbohydrate interactions
MD-2	1	-	1	Lipid-recognition domain
Ribonuclease/ribotoxin;	1	-	1	Extracellular guanyl-specific ribonuclease
Ribonuclease Inhibitor	1	-	1	Enzyme that inhibits RNase activity
Fungal calcium binding	1	-	1	Involved in events where calcium is a second messenger
Chitin biosynthesis protein CHS5	1	-	1	Found at the N-terminus of fungal chitin biosynthesis protein CHS5. It functions as a dimerization domain
Fungalysin (M36)/Thermolysin signature	1	-	1	Metallopeptidase
Lipase (class 3)	1	-	1	Triacylglycerol lipase
Tetratricopeptide repeat domain	-	1	1	Module for protein interaction and mediators for multiprotein complex
Cystatin/monellin	-	1	1	Cysteine protease inhibitors
RuvA domain	-	1	1	Domain related to prokaryotic proteins; DNA helicase that binds DNA at Holliday junction and promotes ATP-dependent branch migration on the hetero-duplex

**Table 5:** Classification of fungal and oomycete effectors with respect to functional domains present.

Database	Protein Sequences	Domain	No Domain
Fungi	228	99 (65 C, 34 NC)	129 (65 C, 64 NC)
Oomycetes	86	34 (12 C, 22 NC)	52 (52 NC)
Total	317	133 (77 C, 56 NC)	181 (65 C, 116 NC)

C, canonical; NC, non-canonical.

**Table 6:** Sequence motifs found in fungal and oomycete true effectors. Top15 MEME motifs found in true, validated fungal and oomycete effectors.

MEME ID	Num. of Hits in the Positive db *	Width	E-Value	Best Possible Match
**Fungal positive database**				
MEME-1	7	50	4.60 × 10^−143^	GHNTDGFDIGSSNHITIDGAHVYNQDDCMAINSGTNITFTNGYCSGGHGL
MEME-2	6	49	8.30 × 10^−98^	DGTRVIFEGRTTFGYQEWEGPLISISGKNIKVKGAPGNKIDGDGARWWD
MEME-3	7	50	4.60 × 10^−98^	NVTYEDITLSEISKYGIVVQQDYKNGKPTGTPTTGVPITNITFNKVTGNV
MEME-4	7	40	2.80 × 10^−61^	SIGSVGGRSDNTVKDVHIANSKVTKSMNGVRIKTVAGATG
MEME-5	4	50	2.40 × 10^−58^	YDNVPVTLKKQGIIAKNAYSLYLNSPDAATGQIIFGGVDNAKYSGSLIAL
MEME-6	4	50	8.50 × 10^−55^	QPYDKCQLLFGVNDANILGDNFLRSAYIVYDLDDNEISLAQVKYTSASNI
MEME-7	4	50	8.30 × 10^−51^	PFSIEYGDGSSSQGTWYKDTVGFGGISIKKQQFADVTSTSIDQGILGIGY
MEME-8	173	8	1.60 × 10^−43^	MKFFTILL
MEME-9	4	41	1.60 × 10^−40^	KRQAVPVTLINEQVSYAADITVGSNKQKLNVIIDTGSSDLW
MEME-10	4	50	3.10 × 10^−37^	YLAPMYKGKLAFDYPPDDGEIDFLFEQIFNKYGQQWFSELHQQHPRWHRG
MEME-11	2	50	8.33 × 10^−28^	ICQQYNANFRFNSGFCSGKDRRWDCYDLNFPTTQSERRVQRRRVCRGEHQ
MEME-12	2	50	5.13 × 10^−27^	QFYDQDNGDYEYFNLSEICDRYQEQDGTVVIEHILVNDRQGRACAMMMIK
MEME-13	4	37	8.40 × 10^−27^	CKDTSKGQTYVRGAWHGGKYGIMYAWYMPKDQPATGN
MEME-14	6	29	4.00 × 10^−27^	AAQAIQKKTSCSTITLRNLKVPAGKTLDL
MEME-15	6	39	3.70 × 10^−36^	GNSEITNLNILNWPVHCFSINHAEGLTIFNINIDNSAGD
**Oomycete positive database**				
MEME-1	3	50	8.20 × 10^−29^	SFQGCADDSGFSLLYSTALPDDDQYVKMCASDNCKSLIESVASLNPPNCD
MEME-2	59	11	1.00 × 10^−29^	RHLRSHYQDEE
MEME-3	28	22	9.90 × 10^−19^	LYEHWHMRGCTPEHVYTILKLN
MEME-4	2	31	9.50 × 10^−10^	CPEMCLDVYDPVGDGEGNEYSNQCYMEMAKC
MEME-5	36	14	1.70 × 10^−16^	MRLCYFLFVAAAAI
MEME-6	2	39	1.30 × 10^−7^	CCDMVCPDNEAPVCGSDGERYPNPCELGITACEHPEQNI
MEME-7	7	49	4.00 × 10^−7^	SPQFQQWMDYISHYNKENPTMQTSLYAALTTHYGDEEMANMLVEAMHSP
MEME-8	3	21	4.30 × 10^−6^	MVKLYCAVVGVAGSAFPVDID
MEME-9	2	43	5.00 × 10^−5^	GGGIIPVGQKTYSVGIRSTAGGDTFCGGALISPTHVLTTTMCT
MEME-10	2	40	7.90 × 10^−5^	FAPVKLPKADGSDIKPGMWSKAMGWGWTSFPNGARANEMQ
MEME-11	2	36	4.00 × 10^−3^	CNCVYVIGPSEVCAGGEEGKDKCVGDTGGPLIKENG
MEME-12	3	50	6.30 × 10^−5^	PCSGLCLNVVDLTCGFSGKCSSSSCTSNTASCAATSGTTEAPAATCAAPT
MEME-13	7	9	8.50 × 10^−3^	PVFNIWLEY
MEME-14	3	39	1.20 × 10^−1^	SPLQRTDEVQHQPDVDDKTNRFLTSEDKDLPLLVTSDGY
MEME-15	2	30	1.30 × 10^−1^	WVAVGTHYVNGTKDGEQLKVIQAQNHTDFN

* Database.

**Table 7:** Validation of WideEffHunter for prediction of fungal and oomycete effector proteins and comparison with EffectorP 3.0, EffectorP 2.0, EffectorP 1.0, and EffectorO.

**WideEffHunter**										
**Data**	**Proteins type**	**Total proteins**	**Results**	**Prediction**	**Sen/Rec**	**Spe**	**PPV/Prec**	**ACC**	**FPR**	**F1 score**
Set 1	Fungi	228	228	1859	1	0.658	0.168	0.68	0.341	0.287
Set 2	Oomycete	86	86							
Set 3	Negatives	4528	1545							
Set 3	Negatives	4528	192	506	1	0.957	0.62	0.96	0.042	0.765
**EffectorP 3.0**										
**Data**	**Proteins type**	**Total proteins**	**Results**	**Prediction**	**Sen/Rec**	**Spe**	**PPV/Prec**	**ACC**	**FPR**	**F1 score**
Set 1	Fungi	228	184	476	0.845	0.952	0.557	0.945	0.047	0.669
Set 2	Oomycete	86	79							
Set 3	Negatives	4528	213							
**EffectorP 2.0**										
**Data**	**Proteins type**	**Total proteins**	**Results**	**Prediction**	**Sen/Rec**	**Spe**	**PPV/Prec**	**ACC**	**FPR**	**F1 score**
Set 1	Fungi	228	153	243	0.564	0.985	0.736	0.958	0.014	0.638
Set 2	Oomycete	86	26							
Set 3	Negatives	4528	64							
**EffectorP 1.0**										
**Data**	**Proteins type**	**Total proteins**	**Results**	**Prediction**	**Sen/Rec**	**Spe**	**PPV/Prec**	**ACC**	**FPR**	**F1 score**
Set 1	Fungi	228	142	255	0.579	0.983	0.713	0.957	0.016	0.639
Set 2	Oomycete	86	40							
Set 3	Negatives	4528	73							
**EffectorO**										
**Data**	**Proteins type**	**Total proteins**	**Results**	**Prediction**	**Sen/Rec**	**Spe**	**PPV/Prec**	**ACC**	**FPR**	**F1 score**
Set 1	Fungi	228	97	961	0.573	0.827	0.187	0.811	0.172	0.281
Set 2	Oomycete	86	83							
Set 3	Negatives	4528	781							

Set 1, validated fungal effectors; Set 2, validated oomycete effectors; Set 3, negative dataset, taken from Carreón-Anguiano et al. (2020) [12]. Sen/Rec: Sensitivity/Recall; Spe: Specificity; PPV/Prec: Positive Predictive Value/Precision; ACC: Accuracy; FPR: False positive rate; F1 score: Measure of the success of binary classifier (score reaches its best value at 1, and worst score at 0).

**Table 8:** Effectoromes predicted by WideEffHunter in selected fungi and oomycetes and comparison with other predictors.

Species	Proteome	Effector Prediction in Reference	Reference	Criteria for Effector Prediction	WideEffHunter ^1^	WideEffHunter ^2^	EffectorP 1.0	EffectorP 2.0	EffectorP 3.0	EffectorO
*Puccinia triticina*	15,685	904	[15]	Motifs	4334	**2805**	4162	2570	7488	11,782
*Venturia inaequalis*	13,233	1369	[42]	Homology to known effectors	3847	**2158**	2744	1832	5524	8968
*Phytophthora infestans*	17,797	5814	[27]	Motif- search and lineage-specific phylogenetic distribution	7143	**3811**	4749	3091	8879	11,952
*Bremia lactucae*	10,102	1777	[27]	Motif- search and lineage-specific phylogenetic distribution	3317	**1812**	2435	1625	4884	6355
*Trichoderma harzianum*	14,095	307	[12]	Size ≤400 amino acids, SP, No TMD, ≥4 Cys	4935	**2693**	2893	1772	4900	8318
*Pestalotiopsis fici*	15,413	381	[43]	Small secreted cysteine-rich proteins, with no conserved domain, with nuclear localization signal (NLS), and repeated sequences (Repeat-containing proteins, or (RCPs)	5201	**2524**	1907	1236	4488	9319
*Xylona heveae*	8205	84	[43]	Small secreted cysteine-rich proteins, with no conserved domain, with nuclear localization signal (NLS), and repeated sequences (Repeat-containing proteins, or (RCPs)	2828	**1517**	1322	756	2819	5680

^1^ Before filtering hits with MEME motifs found in the negative dataset; ^2^ After filtering hits with MEME motifs found in the negative dataset.

## Data Availability

https://github.com/Gisel-Carreon?tab=repositories.

## Supplementary Materials

The following supporting information can be downloaded at: https://www.mdpi.com/article/10.3390/ijms232113567/s1.
