# Supplementary material for: WideEffHunter: An Algorithm to Predict Canonical and Non-Canonical Effectors in Fungi and Oomycetes
Source: Int J Mol Sci. 2022 Nov 5;23(21):13567. doi: 10.3390/ijms232113567 (PMC9653874; doi:10.3390/ijms232113567)
Supplement: Supplementary file 1 [file ijms-23-13567-s001.zip › Table S3. Top15 MEME motifs found in the negative dataset.pdf]

Table S3. Top15 MEME motifs found in the negative dataset

MEME ID	Number of hits in the negative database	Width	E-value	Best possible match
MEME-1	2148	21	5.3×10^{-405}	VKPGEKTAIMGPNGCGKSTLL
MEME-2	2165	21	5.9×10^{-327}	NGRGLSGGQKQRVSIARALYS
MEME-3	2137	29	2.0×10^{-402}	PKILFLDEPTSGLDSETEWNIQRFLRKEF
MEME-4	579	50	2.3×10^{-362}	YCPQTPWIQNMTIRDNILFGSPYDEEWYWQVIHACCLEPDLEML PHGDQT
MEME-5	938	39	1.10×10^{-244}	FRFYEPTSGHITIDGHDISKIPRHDLRSRIGIVPQDPVL
MEME-6	1337	22	4.20×10^{-210}	LCVPPGEMTVVVGPNGCGKSTL
MEME-7	1690	21	6.60×10^{-182}	FDKICVMDEGKVVEYGPHKEL
MEME-8	643	41	2.50×10^{-217}	ASKKLHQDLLRTVMHAPMRFFDTTPTGRIMNRFSQDMDLID
MEME-9	841	21	1.10×10^{-132}	GKNLSQGQRQLLCLARAMVRR
MEME-10	885	29	7.00×10^{-140}	RPDICCLDDPFSALDHHTERHIWDNCFCG
MEME-11	533	29	5.50×10^{-131}	PGTVRSNLDPFHEHTDEECWDALRRVHLW
MEME-12	373	32	1.90×10^{-108}	PDDHSFQRKTGYCEQEDVHLPTLTVRETLEFS
MEME-13	69	50	3.20×10^{-100}	WWGRMKEAQDRQKAHMEATIRNNMKAGKKNNDDNKLRQAK SRQKKLDDRW
MEME-14	580	21	2.60×10^{-85}	KSPLYSHFGETLSGLTTIRAF
MEME-15	851	15	1.80×10^{-79}	RTIICIAHRLSTIMD

Negative dataset was taken from Carreón-Anguiano et al. (2020). It comprises 4528 protein sequences with different length, presence/absence of signal peptide and TMDs
