# Supplementary material for: WideEffHunter: An Algorithm to Predict Canonical and Non-Canonical Effectors in Fungi and Oomycetes
Source: Int J Mol Sci. 2022 Nov 5;23(21):13567. doi: 10.3390/ijms232113567 (PMC9653874; doi:10.3390/ijms232113567)
Supplement: Supplementary file 1 [file ijms-23-13567-s001.zip › Table S5.1 Regular expressions for effector motifs.pdf]

**Table S5.1.** Regular expressions for effector motifs, obtained from several references

| Motif | Regular expression | Reference |
|---|---|---|
| RXLR | (^\w{1,40}\w{1,96}[RKH]\w[LIMFYWK][RALQGTF])*[ED][ED][KR] | Huang et al., 2022; Zhao et al., 2020; Liu et al., 2019; Sonah et al., 2016 |
| Crinkler | (^\w{10,40}\w{1,96}L[FYRL][LKF][ATVRK][KRN]) | Huang et al., 2022; Zhao et al., 2020; Liu et al., 2019 |
| [SG]PC[KR]P | (^\w{10,40}\w{1,96}[GS]PC[KR]P) | Huang et al., 2022; Sperschneider et al., 2013 |
| [WYF]CxTYxSTYL | [WYF]C\wTY\wSTYL | Sperschneider et al., 2013 |
| YFWxC | (^\w{10,40}\w{1,30}[YFW]\wC) | Huang et al., 2022; Zhao et al., 2020; Liu et al., 2019; Xia et al., 2017; Sonah et al., 2016 |
| CFEM 2 | P\wC[AG]\w{2}C\w{8,12}C\w{1,3}[\w,T]D\w{2,5}C\wC\w{9,14}C\w{3,4}C\w{15,16} | Wang et al., 2022; Zhao et al., 2020; Liu et al., 2019 |
| LysM | Y\w{4}G\w{2}[ACFGHIKLMRTVWY] | Zhao et al., 2020; Liu et al., 2019 |
| EAR 1 | L\wL\wL | Zhao et al., 2020; Liu et al., 2019 |
| EAR 2 | DLN\w{2}P | Zhao et al., 2020; Liu et al., 2019 |
| DPBB 1 (PNPi) | T[ACDGNPSTV]ALS[ST]ALF[ACDGNPSTV]{2}G\w[ACDGNPSTV]CG[AGS]C[FHWY][CDEHKNQRST][ILV][ACFGHIKLMRTVWY]C\w{3}[ACDEGHKNQRST][ACDGNPSTV]{2}[CDEHKNQRST][ACFGHIKLMRTVWY]C[ACFGHIKLMRTVWY][ACDGNPSTV]\w{2}[ACDGNPSTV]{2}[ILV][ACFGHIKLMRTVWY][ILV]TATN[ACFGHIKLMRTVWY]CPP[ACDGNPSTV]\w[ACDEGHKNQRST]\w[ACDGNPSTV]{3}\w{2}[ACDGNPSTV]WCNPP[CDEHKNQRST]{2}HFD[ACFGHIKLMRTVWY][AGS]\wP[ACFGHIKLMRTVWY][FHWY]\w[CDEHKNQRST][ILV]A\w{2}[HKR]AG[ILV]VP[ILV][ACDEGHKNQRST][FHWY] | Zhao et al., 2020; |
| CHxC | CH\wC | Huang et al., 2022; |
| G[IFY][ALST]R | G[IFY][ALST]R | Huang et al., 2022; Xia et al., 2017 |
| [LI]xAR | [LI]\wAR | Huang et al., 2022; |
| [RK]VY[LI]R | [RK]VY[LI]R | Huang et al., 2022; |
| YxSL[RK] | Y\wSL[RK] | Huang et al., 2022; Xia et al., 2017 |
| ToxA | RGD | Huang et al., 2022; Zhao et al., 2020; Liu et al., 2019 |
| PDI | [KRHQSA][DENQ]EL | Sonah et al., 2016 |
| YxSL[RK] | Y\wSL[RK] | Adhikari et al., 2013 |
| RSIVEQD | RSIVEQD | Sonah et al., 2016 |
| [LI]xAR[SE][DSE] | [LI]\wAR[SE][DSE] | Xia et al., 2017 |
| [RK]CxCX{12}H | [RK]C\w{2}C\w{12}H | Xia et al., 2017 |
