# Supplementary material for: WideEffHunter: An Algorithm to Predict Canonical and Non-Canonical Effectors in Fungi and Oomycetes
Source: Int J Mol Sci. 2022 Nov 5;23(21):13567. doi: 10.3390/ijms232113567 (PMC9653874; doi:10.3390/ijms232113567)
Supplement: Supplementary file 1 [file ijms-23-13567-s001.zip › Table S5.2. Regular expressions for consensus alignment domains.pdf]

Table S5.2. Regular expressions for consensus alignment domains from SMART, found in fungal or oomycete effectors

Domain	Regular expression
Glycosil hydrolase 10	[ACFGHIKLMRTVWY]K\w{0,3}[ACFGHIKLMRTVWY][CDEHKNQRST][ACDGNPSTV][CDEHKNQRST]{0,2}[ACDGNPSTV]\w{0,27}[ACFGHIKLMRTVWY][ACDGNPSTV][FHWY][CDEHKNQRST]\w{0,4}[ACDGNPSTV]D[ACDEGHKNQRST]\w[ACFGHIKLMRTVWY]{0,2}[CDEHKNQRST][ACFGHIKLMRTVWY][ACDGNPSTV][ACDEGHKNQRST][CDEHKNQRST]{0,2}[ACDGNPSTV][ACFGHIKLMRTVWY]\w{0,3}[ACFGHIKLMRTVWY][HKR]GH[ACDGNPSTV][ILV]{0,2}W[DEHKR][ACDGNPSTV]\w{0,2}Q\wP[ACDEGHKNQRST]W[ACFGHIKLMRTVWY]\w{0,23}[ACDEGHKNQRST]\w[ACFGHIKLMRTVWY]{0,2}[CDEHKNQRST][ACFGHIKLMRTVWY]{0,2}[CDEHKNQRST]{0,2}[FHWY][ILV][CDEHKNQRST][ACDGNPSTV][ILV][ACFGHIKLMRTVWY][ACDEGHKNQRST][HKR][FHWY][ACDEGHKNQRST][ACDGNPSTV][CDEHKNQRST]\w{0,3}[ACFGHIKLMRTVWY][ACDEGHKNQRST]\w{0,10}WDVVN\wE[ACFGHIKLMRTVWY]{0,2}[ACDEGHKNQRST]{0,2}[ACDGNPSTV]\w{0,12}[ACDEGHKNQRST]\w{0,18}[ACDGNPSTV]\w{0,7}[FHWY]\w[CDEHKNQRST][ACFGHIKLMRTVWY]{0,2}G\w{0,11}[CDEHKNQRST][FHWY][ILV]\w{0,2}AF[CDEHKNQRST][ACFGHIKLMRTVWY]A[HKR][ACDEGHKNQRST][ACFGHIKLMRTVWY][ACDGNPSTV]P\w{0,11}[ACDGNPSTV][ACFGHIKLMRTVWY]L[ACFGHIKLMRTVWY]{0,2}NDY[ACDGNPSTV][ACFGHIKLMRTVWY]\w[ACDEGHKNQRST]\w{0,5}[HKR][ACDEGHKNQRST][CDEHKNQRST]\w{0,3}[ACFGHIKLMRTVWY]{0,2}[CDEHKNQRST][ACFGHIKLMRTVWY][ILV][CDEHKNQRST]{0,2}[ACFGHIKLMRTVWY]{0,2}[ACDEGHKNQRST]{0,2}[ACDGNPSTV][ACFGHIKLMRTVWY]\w{0,8}[ILV][DEHKR][AGS][ILV]G\w{0,20}[ACFGHIKLMRTVWY]Q[ACDGNPSTV]H[ACFGHIKLMRTVWY]\w{0,20}[ACDEGHKNQRST]\w{0,6}[ACFGHIKLMRTVWY][CDEHKNQRST][ACDEGHKNQRST][ACDGNPSTV][ILV][CDEHKNQRST][ACFGHIKLMRTVWY]{0,2}[AGS][ACDEGHKNQRST][ACFGHIKLMRTVWY]\w{0,6}[ILV]\w[ILV]TE[ILV]D[ILV][ACDEGHKNQRST][ACFGHIKLMRTVWY]\w{0,36}[CDEHKNQRST][ACDGNPSTV][ACDEGHKNQRST]\w{0,11}[ACFGHIKLMRTVWY]\w[CDEHKNQRST][ACFGHIKLMRTVWY]{0,2}[CDEHKNQRST]\w[ACFGHIKLMRTVWY]{0,2}[ACDEGHKNQRST]\w{0,10}[ACFGHIKLMRTVWY]\w[AGS][ILV][ACDGNPSTV][ACFGHIKLMRTVWY]WG[ACFGHIKLMRTVWY]\w[DE][ACDEGHKNQRST]\w{0,3}[ACDGNPSTV]W[ACFGHIKLMRTVWY]\w{0,34}[ACDGNPSTV][ACFGHIKLMRTVWY][ILV][ACFGHIKLMRTVWY][ACDGNPSTV][ACDEGHKNQRST][CDEHKNQRST][ACFGHIKLMRTVWY][CDEHKNQRST][ACDGNPSTV][HKR]\w[ACDGNPSTV][ACFGHIKLMRTVWY]\w[ACFGHIKLMRTVWY]{0,2}\w{0,2}
Glycosil hydrolase 25	\w{0,2}[AGS][ACFGHIKLMRTVWY]\w[ACFGHIKLMRTVWY]\w[ACDGNPSTV][FHWY][CDEHKNQRST]\w{0,7}[ACDEGHKNQRST][ACFGHIKLMRTVWY][ACDGNPSTV][ACDEGHKNQRST]\w[ACDEGHKNQRST][CDEHKNQRST][ACDGNPSTV]{0,2}[ACFGHIKLMRTVWY]\w{0,3}[ACFGHIKLMRTVWY]{0,4}[CDEHKNQRST][ACFGHIKLMRTVWY]{0,2}[ACDEGHKNQRST][ACDGNPSTV][ACDEGHKNQRST][ACDGNPSTV][FHWY][ACFGHIKLMRTVWY][ACDGNPSTV]\w[ACFGHIKLMRTVWY][FHWY][ACDGNPSTV]\w[ACDEGHKNQRST]\w{0,5}[CDEHKNQRST][ACFGHIKLMRTVWY][ACDGNPSTV]\w{0,3}[ACFGHIKLMRTVWY]{0,3}[ACDEGHKNQRST][ACFGHIKLMRTVWY]\w[CDEHKNQRST]\w{0,4}[ACFGHIKLMRTVWY][ACDEGHKNQRST]\w[ACDGNPSTV]\w{0,7}[ACDGNPSTV][ACDEGHKNQRST]\w[CDEHKNQRST][ACFGHIKLMRTVWY][CDEHKNQRST][FHWY]F\w[CDEHKNQRST]\w[ACDGNPSTV][ACDEGHKNQRST]\w{0,3}[ACFGHIKLMRTVWY][CDEHKNQRST]\w{0,2}[ACDGNPSTV]\w{0,3}[ACFGHIKLMRTVWY]{0,2}[ACDGNPSTV][ILV][DE][ACFGHIKLMRTVWY][CDEHKNQRST]\w[ACDGNPSTV]\w{0,2}[ACDEGHKNQRST][ACFGHIKLMRTVWY]\w{0,2}[ACDGNPSTV]\w
Glycosil hydrolase 32	H[ACFGHIKLMRTVWY][CDEHKNQRST]P\w[CDEHKNQRST][ACDGNPSTV][ACFGHIKLMRTVWY]{0,2}NDPNG\wNDPNG{0,3}\w{0,2}[CDEHKNQRST]G[ACFGHIKLMRTVWY]YH[ILV]FYQ[FHWY][CDEHKNQRST]P\w[ACDGNPSTV]{0,2}\w{0,4}[ACFGHIKLMRTVWY]\w{0,4}[

	ACFGHIKLMRTVWY]{0,2}W[AGS]H[ACDGNPSTV]{0,2}S\w{0,4}[CDEHKNQRST]D[ACFGHIKLMRTVWY][ILV][CDEHKNQRST]W\w{0,4}[ACDEGHKNQRST]\w{0,3}A[ACFGHIKLMRTVWY]{0,2}P[CDEHKNQRST][ACDEGHKNQRST]\w{0,5}[DEHKR]\w[ACDEGHKNQRST]G\w{0,4}[ACDGNPSTV][FHWY][ST]GS[ACDGNPSTV]{0,2}\w{0,3}[ACDGNPSTV][ACDEGHKNQRST]\w{0,4}[ACFGHIKLMRTVWY]{0,2}[FHWY][ST][AGS]\w{0,14}[ACFGHIKLMRTVWY][ACDEGHKNQRST]\w{0,2}[ACDEGHKNQRST]\w{0,19}[ACDEGHKNQRST]{0,2}\w[ACDEGHKNQRST]\w{0,2}[ACFGHIKLMRTVWY]\w[ACFGHIKLMRTVWY][ACDGNPSTV]\w[ACDGNPSTV]\w[ACFGHIKLMRTVWY]{0,2}[CDEHKNQRST][FHWY]\wK\w{0,4}[ACDGNPSTV]{0,2}[ILV][ACFGHIKLMRTVWY]\w{0,5}[ACDGNPSTV]\w{0,13}[ACDEGHKNQRST][CDEHKNQRST]FRDP[CDEHKNQRST][ACDGNPSTV][ACFGHIKLMRTVWY]\w{0,2}[ACFGHIKLMRTVWY]\w[ACDEGHKNQRST]\w{0,8}[CDEHKNQRST]\wW[ACFGHIKLMRTVWY]{0,3}[ILV][AGS]\w[ACDEGHKNQRST][CDEHKNQRST]\w{0,10}[ACFGHIKLMRTVWY]{0,3}Y[CDEHKNQRST][ST]\w{0,13}[ACDGNPSTV][ACFGHIKLMRTVWY]{0,2}\w[CDEHKNQRST][FHWY][CDEHKNQRST][ACFGHIKLMRTVWY]\w{0,5}[ACFGHIKLMRTVWY][ACDEGHKNQRST]\w{0,8}[ACFGHIKLMRTVWY][AGS]\w{0,8}[ACFGHIKLMRTVWY][FHWY]EC[ACDGNPSTV]{0,2}[ACFGHIKLMRTVWY]{0,2}\w[ILV][ACDGNPSTV]\w*[ACFGHIKLMRTVWY]V[ACFGHIKLMRTVWY]{0,3}[ACDGNPSTV]\w[CDEHKNQRST]\w*[CDEHKNQRST][ACFGHIKLMRTVWY]Y\w[ACFGHIKLMRTVWY]G\w[ACFGHIKLMRTVWY][ACDGNPSTV]\w{0,11}[ACDEGHKNQRST][FHWY]\w[ACDGNPSTV]\w*[ACFGHIKLMRTVWY]D[ACFGHIKLMRTVWY]G\w[CDEHKNQRST][FHWY]YA[ACDGNPSTV][CDEHKNQRST]\w{0,2}[ST][FHWY]\w[ACDGNPSTV]\w{0,10}[ACDEGHKNQRST]\w[DEHKR][ACDEGHKNQRST][ILV][ACFGHIKLMRTVWY]{0,2}[AGS]W[ACFGHIKLMRTVWY][ACDGNPSTV]\w{0,2}[CDEHKNQRST]\w{0,3}[ACDEGHKNQRST]{0,2}[CDEHKNQRST][ACFGHIKLMRTVWY]\w{0,6}[CDEHKNQRST][ACDGNPSTV]W[ACDEGHKNQRST][ACDGNPSTV][ACFGHIKLMRTVWY]\w[ACDGNPSTV][ACFGHIKLMRTVWY][ACDGNPSTV]R[CDEHKNQRST][ILV]\w{0,14}[ACDEGHKNQRST]{0,2}[CDEHKNQRST]\w{0,9}[ILV][ACFGHIKLMRTVWY][CDEHKNQRST][ACFGHIKLMRTVWY]P[ILV]\w[CDEHKNQRST][ACFGHIKLMRTVWY][CDEHKNQRST][ACDEGHKNQRST]L[CDEHKNQRST]\w{0,20}[ACFGHIKLMRTVWY]\w[ACFGHIKLMRTVWY][ACDEGHKNQRST]\w{0,14}[CDEHKNQRST]\w{0,2}[DE][ILV]\w[ACFGHIKLMRTVWY][CDEHKNQRST][ACFGHIKLMRTVWY][ACDEGHKNQRST]\w[ACDEGHKNQRST]\w*[ACFGHIKLMRTVWY][ACDGNPSTV][ILV][ACFGHIKLMRTVWY]{0,3}[ACDEGHKNQRST]\w{0,7}[ACDEGHKNQRST]\w[ACFGHIKLMRTVWY]\w[ILV][ACFGHIKLMRTVWY]{0,2}\w[ACFGHIKLMRTVWY]\w[CDEHKNQRST]\w{0,17}[ACFGHIKLMRTVWY]{0,2}\w[DE][CDEHKNQRST]\w{0,2}[CDEHKNQRST][ACDEGHKNQRST]\w{0,10}[ACDEGHKNQRST]\w{0,3}[ACFGHIKLMRTVWY]\w{0,2}[ACFGHIKLMRTVWY][ACDEGHKNQRST][ACDGNPSTV]\w{0,3}[ACFGHIKLMRTVWY]\w{0,5}[ACDEGHKNQRST]\w{0,8}[ACFGHIKLMRTVWY][CDEHKNQRST][ACFGHIKLMRTVWY][CDEHKNQRST][ACFGHIKLMRTVWY]{0,2}[ILV]D[CDEHKNQRST][AGS]\w[ILV]E\wF[ACFGHIKLMRTVWY][ACDEGHKNQRST]{0,2}G
Pectin lyase fold	\w[ACFGHIKLMRTVWY]{0,3}\w[ACFGHIKLMRTVWY]\w{0,7}[ACFGHIKLMRTVWY]\w[ACFGHIKLMRTVWY][ACDGNPSTV]\w*[AGS]\w[CDEHKNQRST][ST][ILV]\wG\w[ACDGNPSTV]{0,2}[CDEHKNQRST][ACDGNPSTV][ACDEGHKNQRST][ILV]\w[ACDEGHKNQRST]\w{0,2}[ACFGHIKLMRTVWY][ACDEGHKNQRST][ILV][ACFGHIKLMRTVWY]{0,2}\w{0,2}[ACDGNPSTV][CDEHKNQRST]\w{0,3}[ACDGNPSTV][ILV]{0,3}[CDEHKNQRST][ACDGNPSTV][ILV][ACDEGHKNQRST][ACFGHIKLMRTVWY][CDEHKNQRST]{0,2}\w{0,23}[ACDEGHKNQRST]\wD[AGS][ILV][ACDEGHKNQRST][ILV]\w{0,7}[AGS][CDEHKNQRST]{0,3}[ILV]W[ILV]DH[ACDGNPSTV][CDEHKNQRST][ACFGHIKLMRTVWY]\w[ACDEGHKNQRST]{0,2}\w{0,18}D[AGS][ACFGHIKLMRTVWY]{0,2}D[ACFGHIKLMRTVWY]{0,2}\w[ACDGNPSTV][AGS][ACDGNPSTV][ACFGHIKLMRTVWY][ILV]T[ILV]S\w[ACDGNPSTV][ACFGHIKLMRTVWY]{0,2}[CDEHKNQRST]{0,2}[FHWY][CDEHKNQRST][ACFGHIKLMRTVWY][ACDGNPSTV]\wL[ACFGHIKLMRTVWY]G[ACDEGHKNQRST][ACDGNPSTV]{0,3}\w{0,4}[ACDEGHKNQRST]\w{0,7}

	[ILV]T[ACFGHIKLMRTVWY][ACDEGHKNQRST]\wN[ACFGHIKLMRTVWY][FHWY][ACDEGHKNQRST][CDEHKNQRST]\w[ACFGHIKLMRTVWY]\w[ACDEGHKNQRST]R[ACFGHIKLMRTVWY]P[ACDEGHKNQRST][ACFGHIKLMRTVWY]R[ACFGHIKLMRTVWY][AGS]\w{0,2}[ACFGHIKLMRTVWY]H[ILV][ACFGHIKLMRTVWY]N{0,2}\w[FHWY][ACDEGHKNQRST]{0,2}\w[ACDEGHKNQRST]\w{0,13}[ACFGHIKLMRTVWY][AGS][ACFGHIKLMRTVWY][ACDGNPSTV]{0,2}[ACDEGHKNQRST]\w[ACDEGHKNQRST][ACDGNPSTV][CDEHKNQRST][ILV][ACFGHIKLMRTVWY][ACDGNPSTV][CDEHKNQRST][ACDGNPSTV]N[ACFGHIKLMRTVWY]F\w[ACDEGHKNQRST][ACDGNPSTV][ACDEGHKNQRST]
RlpA	T[ACDGNPSTV]ALS[ST]ALF[ACDGNPSTV]{0,2}G\w[ACDGNPSTV]CG[AGS]C[FHWY][CDEHKNQRST][ILV][ACFGHIKLMRTVWY]C\w{0,3}[ACDEGHKNQRST][ACDGNPSTV]{0,2}[CDEHKNQRST][ACFGHIKLMRTVWY]C[ACFGHIKLMRTVWY][ACDGNPSTV]\w{0,2}[ACDGNPSTV]{0,2}[ILV][ACFGHIKLMRTVWY][ILV]TATN[ACFGHIKLMRTVWY]CPP[ACDGNPSTV]\w[ACDEGHKNQRST].\w.[ACDGNPSTV]{0,3}\w{0,2}[ACDGNPSTV]WCNPP[CDEHKNQRST]{0,2}HFD[ACFGHIKLMRTVWY][AGS]\wP[ACFGHIKLMRTVWY][FHWY]\w[CDEHKNQRST][ILV]A\w{0,2}[HKR]AG[ILV]VP[ILV][ACDEGHKNQRST][FHWY]
CFEM	\w[ACDEGHKNQRST]\w[ACFGHIKLMRTVWY]W\w{0,13}[ILV][ACDGNPSTV][FHWY][ACFGHIKLMRTVWY]{0,2}\w{0,10}[ACDEGHKNQRST]\w[ACFGHIKLMRTVWY][ACDGNPSTV]\w{0,17}[CDEHKNQRST][ACDEGHKNQRST][ACFGHIKLMRTVWY]{0,2}[ACDEGHKNQRST][CDEHKNQRST]\w[AGS][ACFGHIKLMRTVWY][CDEHKNQRST]\w[FHWY][CDEHKNQRST]{0,2}\w[ACDGNPSTV]\w{0,11}[ACDGNPSTV][ILV][CDEHKNQRST]F\w{0,18}[ACDEGHKNQRST]{0,3}\w[ACFGHIKLMRTVWY][ILV]\w[ACFGHIKLMRTVWY]{0,2}[ACDEGHKNQRST]\w[ACDEGHKNQRST]\w{0,12}[ACFGHIKLMRTVWY][ACDGNPSTV]\w{0,17}[AGS][ACFGHIKLMRTVWY]{0,4}\w{0,13}[ACDEGHKNQRST]{0,2}\w[ACFGHIKLMRTVWY][CDEHKNQRST]\w{0,7}[ACDEGHKNQRST]\w{0,2}[ACDGNPSTV]\w[ACDEGHKNQRST]\w[ACDGNPSTV]\w{0,20}[ACDGNPSTV][ACFGHIKLMRTVWY]{0,2}HE[ACFGHIKLMRTVWY]{0,2}H[ACDGNPSTV][ILV]G[ACFGHIKLMRTVWY]\w{0,2}H[CDEHKNQRST]{0,2}[ACDEGHKNQRST][ACFGHIKLMRTVWY]\w[ACDGNPSTV]\w{0,8}[ACFGHIKLMRTVWY]{0,2}\w{0,3}[ACFGHIKLMRTVWY][CDEHKNQRST]\w{0,11}[ACDEGHKNQRST][ACFGHIKLMRTVWY]\w[ACFGHIKLMRTVWY]\w{0,10}[CDEHKNQRST]\w[ACFGHIKLMRTVWY][ACDEGHKNQRST]\w{0,7}[FHWY][ACDGNPSTV]\w
Chitin binding Peritrophin A domain	\w{0,2}C\w*[ACDEGHKNQRST][ACFGHIKLMRTVWY]{0,2}[ACDGNPSTV]\w*C[CDEHKNQRST][ACDEGHKNQRST]\w[FHWY][ACFGHIKLMRTVWY]\wC\w[ACDEGHKNQRST][ACDGNPSTV]\w*[ACFGHIKLMRTVWY]\w[ACFGHIKLMRTVWY]\wC\w{0,2}[ACDGNPSTV]\w[ACFGHIKLMRTVWY]{0,2}[FHWY][ACDGNPSTV]\w[ACDEGHKNQRST]\w*C\w*C\w{0,2}
Elicitin	[ACDEGHKNQRST]{0,2}C[ACDGNPSTV]{0,3}[ACDEGHKNQRST]\w[ACDEGHKNQRST]{0,2}\w{0,7}[ILV][ACFGHIKLMRTVWY][ACDGNPSTV]{0,3}\w{0,5}[ACDGNPSTV]C[ACDEGHKNQRST][ACDGNPSTV]\w[ACDGNPSTV]\w[ACDEGHKNQRST][ACFGHIKLMRTVWY]\w{0,17}[ACDGNPSTV]\w{0,4}[CDEHKNQRST][ACFGHIKLMRTVWY]\w{0,2}[ACFGHIKLMRTVWY]C[ACDEGHKNQRST][ACDGNPSTV]{0,3}C\w[ACDEGHKNQRST][ACFGHIKLMRTVWY][ILV][ACDEGHKNQRST]{0,2}[ACFGHIKLMRTVWY]\w{0,2}[ACDEGHKNQRST]\w[ACDEGHKNQRST]\w{0,3}[ACDGNPSTV][CDEHKNQRST]C\w[ACFGHIKLMRTVWY]\w{0,8}[ACFGHIKLMRTVWY]\w[ACFGHIKLMRTVWY]\w[ACDEGHKNQRST][CDEHKNQRST]\w[ACFGHIKLMRTVWY][ACDEGHKNQRST]\w[ACFGHIKLMRTVWY]\w[ACDGNPSTV]\wC[ACDGNPSTV]
Nudix Box	[CDEHKNQRST]{0,2}[ACFGHIKLMRTVWY]LDL\w[ACDGNPSTV]RF[ILV]{0,2}[ACDGNPSTV][ILV]P\w[CDEHKNQRST][DE][ACFGHIKLMRTVWY][CDEHKNQRST][ACDGNPSTV][ACFGHIKLMRTVWY]\wR[ILV]F[CDEHKNQRST][ACFGHIKLMRTVWY]E\wA\wW[FHWY]Y\wD[A

	CFGHIKLMRTVWY]{0,3}\w{0,15}[ACDGNPSTV][ACDEGHKNQRST]LP[CDEHKNQRST][ACFGHIKLMRTVWY][ACDEGHKNQRST][ACFGHIKLMRTVWY][CDEHKNQRST][ACDEGHKNQRST]F[ACDGNPSTV]\w{0,14}[ACFGHIKLMRTVWY]{0,2}[CDEHKNQRST]{0,2}[ACDGNPSTV]\w{0,4}P[ACFGHIKLMRTVWY][ILV]\w{0,2}[ACFGHIKLMRTVWY]\w{0,8}[CDEHKNQRST][ACFGHIKLMRTVWY][DEHKR][CDEHKNQRST][ACFGHIKLMRTVWY]{0,2}[ACDEGHKNQRST][CDEHKNQRST][FHWY]\w[CDEHKNQRST]YK
Fungal cellulose binding domain	[ACDGNPSTV]\w[FHWY][AGS]QCGG\w[ACDEGHKNQRST]\w{0,2}[FHWY][ACDGNPSTV]G[ACDGNPSTV][ST]\w[ACFGHIKLMRTVWY]C\w[CDEHKNQRST][ACDGNPSTV]\w[ACFGHIKLMRTVWY]C\w[ACFGHIKLMRTVWY]\w[ACDGNPSTV]\w{0,3}[FHWY]YSQC[ACFGHIKLMRTVWY]
Thiamine binding	G\w{0,39}[ACDEGHKNQRST][ACFGHIKLMRTVWY][ACDEGHKNQRST][ACFGHIKLMRTVWY]\w{0,8}[ACFGHIKLMRTVWY][AGS][ACFGHIKLMRTVWY]{0,2}[ACDGNPSTV][ACFGHIKLMRTVWY][ACDGNPSTV][ACDEGHKNQRST]\w{0,6}[ACFGHIKLMRTVWY][ACDGNPSTV][ACFGHIKLMRTVWY][CDEHKNQRST][ACDGNPSTV][ACFGHIKLMRTVWY][HKR][FHWY][ACDGNPSTV][ILV]\w{0,15}[ACDGNPSTV]\w{0,2}[ACFGHIKLMRTVWY]\w[ACFGHIKLMRTVWY]\w[ACDEGHKNQRST]\w[ACFGHIKLMRTVWY][ACDGNPSTV]\wS\wN[ACDEGHKNQRST][ACFGHIKLMRTVWY]{0,2}\w[ACDEGHKNQRST]{0,2}\w{0,8}[ACFGHIKLMRTVWY][CDEHKNQRST][ACFGHIKLMRTVWY][CDEHKNQRST]{0,2}[ACDGNPSTV]\w{0,2}[ILV]
Cutinase (alpha-beta hydrolase)	[ACDEGHKNQRST][ACDGNPSTV]C[ACDGNPSTV]{0,2}[ILV]\w[ACFGHIKLMRTVWY][ILV][ACFGHIKLMRTVWY][ACDGNPSTV][CDEHKNQRST]G[ST]\w[DE]\w{0,15}[ACDGNPSTV]{0,2}[ACFGHIKLMRTVWY][AGS]\w{0,10}[ACFGHIKLMRTVWY]{0,2}\w[ACDGNPSTV][ACFGHIKLMRTVWY][ACDGNPSTV][CDEHKNQRST][ACDGNPSTV][ILV][CDEHKNQRST][ACDEGHKNQRST]{0,2}[ACFGHIKLMRTVWY]\w{0,6}[ACDGNPSTV][ACDEGHKNQRST][CDEHKNQRST][ACFGHIKLMRTVWY][ACDEGHKNQRST][ACFGHIKLMRTVWY]\w[ACDGNPSTV][ILV][ACDGNPSTV]\w{0,5}Y[ACDGNPSTV]A[ACDGNPSTV]\w[ACDEGHKNQRST]\w{0,23}[ACDEGHKNQRST][ACDGNPSTV][ACFGHIKLMRTVWY][ACDEGHKNQRST]\wG[ACFGHIKLMRTVWY][ACDEGHKNQRST][ACDGNPSTV][ACFGHIKLMRTVWY]\w[ACDEGHKNQRST][ACFGHIKLMRTVWY][ILV][CDEHKNQRST][ACDEGHKNQRST][ACFGHIKLMRTVWY]{0,2}[ACDEGHKNQRST][CDEHKNQRST]C\wP[CDEHKNQRST]T[CDEHKNQRST][ACFGHIKLMRTVWY]V[ILV][ACDGNPSTV]G[FHWY]SQGA\w[ILV][ACFGHIKLMRTVWY][ACDGNPSTV]\w[ACFGHIKLMRTVWY]{0,2}\w{0,18}[ACDEGHKNQRST][ACDGNPSTV][ACFGHIKLMRTVWY][ACDGNPSTV]{0,2}\w{0,3}[CDEHKNQRST]{0,2}[ILV][ACDEGHKNQRST][AGS][ILV][ACDGNPSTV][ACFGHIKLMRTVWY]{0,2}[AGS][CDEHKNQRST][ACDGNPSTV][ACDEGHKNQRST]\w{0,5}[ACDEGHKNQRST][ACFGHIKLMRTVWY]\w{0,40}[ACDEGHKNQRST]\w{0,3}[ACDGNPSTV][ACFGHIKLMRTVWY][ACDGNPSTV]\w{0,13}[ACFGHIKLMRTVWY][ACDGNPSTV]{0,2}[HKR][HKR]\w{0,4}[ACFGHIKLMRTVWY][CDEHKNQRST][ACFGHIKLMRTVWY]C[ACDEGHKNQRST]\w{0,3}[ACDGNPSTV]D\w[ACFGHIKLMRTVWY]C[ACDGNPSTV]\w{0,12}[ACDGNPSTV]\w{0,14}[ACDGNPSTV]H\w{0,4}Y\w{0,2}[ACDEGHKNQRST][ACDGNPSTV]\w{0,4}[ACDEGHKNQRST]{0,2}[AGS][ACFGHIKLMRTVWY][ACDEGHKNQRST][ACFGHIKLMRTVWY]\w{0,3}[ACFGHIKLMRTVWY]{0,2}[ACDEGHKNQRST]{0,2}[ACFGHIKLMRTVWY]
Fungal hydrophobin signature	C[ACDGNPSTV]\w{0,3}[AGS]\w[ILV][CDEHKNQRST]CCN[CDEHKNQRST][ACFGHIKLMRTVWY][ACDEGHKNQRST]{0,2}[ACDGNPSTV]{0,3}\w{0,3}[ACFGHIKLMRTVWY]\w{0,11}L[ILV][ACDGNPSTV][ACFGHIKLMRTVWY][ACDGNPSTV]\w[ACDGNPSTV]{0,2}\w{0,2}[ACFGHIKLMRTVWY]\w{0,3}[ACFGHIKLMRTVWY]{0,2}\w[CDEHKNQRST]C[ACDGNPSTV]{0,2}[ILV][ACDGNPSTV][ACFGHIKLMRTVWY][ILV][ACDGNPSTV][ACFGHIKLMRTVWY][ACDGNPSTV]\w{0,11}[ACDGNPSTV]{0,2}[CDEHKNQRST]C[ACDGNPSTV]{0,2}[CDEHKNQRST]

	[ACDGNPSTV]{0,2}CC[CDEHKNQRST][ACDGNPSTV]{0,2}\w{0,4}[ACDEGHKNQRST]\w[ACDGNPSTV][AGS][ACFGHIKLMRTVWY][ILV][ACDGNPSTV]\w{0,2}[ILV][ACDGNPSTV]C
Chymotrypsin	[CDEHKNQRST][ILV]{0,2}G\w{0,2}G\w{0,6}[ACDGNPSTV][ACDEGHKNQRST]\w[ACDEGHKNQRST][CDEHKNQRST][ACFGHIKLMRTVWY]P[FHWY]\w[ACDGNPSTV][ACDEGHKNQRST][ILV]\w{0,19}[ACFGHIKLMRTVWY]{0,2}CGG[ACDGNPSTV][ILV]{0,2}[ACDGNPSTV]\w[CDEHKNQRST]\w[FHWY][ILV]{0,2}TAAHC[ACFGHIKLMRTVWY]\w[ACDEGHKNQRST]\w{0,19}[ACFGHIKLMRTVWY][CDEHKNQRST][ILV][ACFGHIKLMRTVWY]{0,2}G[ACDEGHKNQRST][ACFGHIKLMRTVWY][ACDEGHKNQRST][ACFGHIKLMRTVWY]\w{0,22}[ACFGHIKLMRTVWY][ACDEGHKNQRST][ACFGHIKLMRTVWY][ACDEGHKNQRST]{0,2}[ACFGHIKLMRTVWY]{0,2}\w{0,4}[DEHKR]\w[ACDEGHKNQRST][FHWY][CDEHKNQRST]\w{0,15}[ACFGHIKLMRTVWY][ACDEGHKNQRST]{0,2}D[ILV][ACFGHIKLMRTVWY]\w{0,7}[ILV]{0,2}[DEHKR][ILV][ACDEGHKNQRST][CDEHKNQRST]\w{0,7}[ACFGHIKLMRTVWY]\w[ACFGHIKLMRTVWY][ACDGNPSTV][ACDEGHKNQRST]\w{0,3}[ILV][ACDEGHKNQRST][ACDGNPSTV][ACFGHIKLMRTVWY][ACDEGHKNQRST]L[ACDGNPSTV][ACDEGHKNQRST]\w*[ACDEGHKNQRST]{0,2}\w[ACDGNPSTV][ACFGHIKLMRTVWY]{0,2}[ACDGNPSTV]GWG\w[ACFGHIKLMRTVWY][ACDEGHKNQRST]\w*[ACDGNPSTV][ACDEGHKNQRST]\wL[CDEHKNQRST]\w{0,8}[ACDEGHKNQRST][ACDGNPSTV][CDEHKNQRST][ILV]\w[ILV][ACFGHIKLMRTVWY][ACDGNPSTV][CDEHKNQRST][ACDEGHKNQRST]\w{0,4}C[ACDEGHKNQRST]{0,2}\w{0,4}[ACFGHIKLMRTVWY]\w*[ACFGHIKLMRTVWY][ACDEGHKNQRST]{0,3}\w[ILV]C[ACDGNPSTV][AGS]\w*[ACDEGHKNQRST][ACFGHIKLMRTVWY][ACDGNPSTV]{0,2}C[CDEHKNQRST]\w*GDSGGP[ILV][ACFGHIKLMRTVWY]{0,2}[ACDEGHKNQRST]\w*[ACFGHIKLMRTVWY]{0,3}G[ILV][ACDGNPSTV]S[FHWY]G\w*C[ACDGNPSTV]\w*[ACDEGHKNQRST][ACFGHIKLMRTVWY]P[AGS][ILV][FHWY][ACDGNPSTV][CDEHKNQRST][ILV][ACDGNPSTV]\w{0,3}[ACFGHIKLMRTVWY]{0,2}[ACDGNPSTV]W[ILV]
Kazal	\wC\w*C[ACDGNPSTV]\w*[ACDEGHKNQRST]\wVCG[ST][DE][ACDGNPSTV]\wTY[ACDGNPSTV]{0,2}\wC\w[ACFGHIKLMRTVWY][CDEHKNQRST][ACFGHIKLMRTVWY][ACDEGHKNQRST]{0,2}[ACFGHIKLMRTVWY]\w*[ACDEGHKNQRST]{0,2}\w[ACDEGHKNQRST][ILV][ACDEGHKNQRST][ACFGHIKLMRTVWY]\w[ACFGHIKLMRTVWY][ACDEGHKNQRST]G\wC
DUF1041	[CDEHKNQRST][ACDEGHKNQRST][ACFGHIKLMRTVWY][ILV][HKR][DEHKR]C[ACFGHIKLMRTVWY][DEHKR][ACDEGHKNQRST][ACDGNPSTV][ACFGHIKLMRTVWY]\w{0,2}[ACDGNPSTV]Y[CDEHKNQRST][ACFGHIKLMRTVWY][ILV]\w[CDEHKNQRST]\w[ACDGNPSTV][ACFGHIKLMRTVWY]\w{0,23}[CDEHKNQRST][ACDEGHKNQRST]\w{0,14}[ACDGNPSTV]{0,2}\w{0,12}[DEHKR][CDEHKNQRST]L[DE]\w[ACFGHIKLMRTVWY]\w[HKR]\w{0,2}L[ACFGHIKLMRTVWY][CDEHKNQRST]L[ACFGHIKLMRTVWY][ILV][CDEHKNQRST][ILV]{0,2}[CDEHKNQRST]{0,2}[ACDGNPSTV][CDEHKNQRST]{0,3}[FHWY][ACDGNPSTV]\w{0,13}[CDEHKNQRST][ACDEGHKNQRST]F[ACDGNPSTV]\w{0,3}[ACDGNPSTV][ILV][ACFGHIKLMRTVWY]\w{0,2}[CDEHKNQRST][AGS][CDEHKNQRST][ACFGHIKLMRTVWY]{0,3}[ACDGNPSTV]L[FHWY][ACDGNPSTV]\wD[ACFGHIKLMRTVWY][DEHKR]\w[ACDGNPSTV]L[CDEHKNQRST]\w[CDEHKNQRST]
DUF1086	\w[ACDGNPSTV]{0,2}[AGS]\w{0,8}[CDEHKNQRST]\w{0,2}[CDEHKNQRST]\w{0,12}[HKR][CDEHKNQRST]{0,2}[ACDEGHKNQRST][CDEHKNQRST][ACDEGHKNQRST][CDEHKNQRST]\w{0,7}[ACDGNPSTV][ILV][ACFGHIKLMRTVWY][ACDEGHKNQRST]{0,2}\w[ACDGNPSTV][ACDEGHKNQRST][CDEHKNQRST][ACFGHIKLMRTVWY][DEHKR]VLGF[ACDGNPSTV]\w[CDEHKNQRST]QR[CDEHKNQRST][ACFGHIKLMRTVWY]F[ACFGHIKLMRTVWY][CDEHKNQRST][ACDGNPSTV][ILV][ACFGHIKLMRTVWY]R[FHWY]G[ACFGHIKLMRTVWY][ACDGNPSTV]{0,2}\w[DE]\w{0,7}W\w{0,2}[ACFGHIKLMRTVWY]{0,2}\w[CDEHKNQRST]L[HKR]\wK[ST]\w[DEHKR][ACDEGHKNQRST][ACFGHIKLMRTVWY][CDEHKNQRST]\w{0,2}[ACDEGHKNQRST]Y[ACDGNPSTV][ACDEGHKNQRST]LF[ACFGHIKLMRTVWY][CDEHKNQRST]H

	[ILV][ACDGNPSTV]E[ACDGNPSTV][ACFGHIKLMRTVWY][ACDGNPSTV][DE]\w{0,2}[AGS]\w[CDEHKNQRST][FHWY][ACDGNPSTV]\w{0,8}DGVP[HKR]EG[ACFGHIKLMRTVWY][CDEHKNQRST][ACFGHIKLMRTVWY][CDEHKNQRST][ACDEGHKNQRST]\w[ILV]L[ACDGNPSTV]R[ILV][AGS][ACFGHIKLMRTVWY]{0,2}\wL[ILV][DEHKR]{0,2}KV[CDEHKNQRST]\w[ACFGHIKLMRTVWY][CDEHKNQRST]{0,2}[ACFGHIKLMRTVWY][ACDGNPSTV]{0,2}[ACDEGHKNQRST]\w{0,2}[ACFGHIKLMRTVWY][ACDGNPSTV][CDEHKNQRST][ACFGHIKLMRTVWY]\w{0,2}
DUF3385	V[ILV][ACDEGHKNQRST]P[ACFGHIKLMRTVWY]\w[CDEHKNQRST][FHWY]P[ACDEGHKNQRST]L{0,2}[ACDEGHKNQRST][ACFGHIKLMRTVWY]L[ACFGHIKLMRTVWY][CDEHKNQRST][ACFGHIKLMRTVWY][ILV][ACDEGHKNQRST][ACDGNPSTV][CDEHKNQRST]\w[CDEHKNQRST]{0,2}\w{0,3}[ILV]R[ACFGHIKLMRTVWY][DE][ACDGNPSTV][ILV][HKR][ACFGHIKLMRTVWY]{0,2}G[ILV]{0,2}GALDP\w{0,5}[FHWY][CDEHKNQRST][ACFGHIKLMRTVWY][CDEHKNQRST]\w{0,53}[ACDEGHKNQRST]\w[ACDEGHKNQRST][CDEHKNQRST]\w{0,9}[ACDEGHKNQRST]{0,3}\w{0,3}[ACDGNPSTV]\w{0,3}[ACFGHIKLMRTVWY]{0,2}[CDEHKNQRST]\w{0,11}[ACDEGHKNQRST]\w[DE]{0,2}[FHWY]{0,2}\w[ACDGNPSTV][ILV][ACDGNPSTV]\w[ILV][ACDGNPSTV]{0,2}L[ACFGHIKLMRTVWY][CDEHKNQRST]\w[ILV]{0,2}\w{0,5}[DEHKR][DE]\w[ACDGNPSTV][ACFGHIKLMRTVWY][ACDGNPSTV][CDEHKNQRST][ACFGHIKLMRTVWY][CDEHKNQRST][ACDEGHKNQRST]\w[ACDGNPSTV]\w[ILV][CDEHKNQRST][AGS][ILV][ACFGHIKLMRTVWY]\w[ILV][ACFGHIKLMRTVWY]\w{0,2}[CDEHKNQRST][ACDGNPSTV]\w[ACDGNPSTV][ACFGHIKLMRTVWY][ACDEGHKNQRST][ACDGNPSTV]\w[ACDGNPSTV]\w[FHWY][ILV][ACDGNPSTV][CDEHKNQRST][ILV][ACFGHIKLMRTVWY]\w{0,3}P[ACDEGHKNQRST][ACFGHIKLMRTVWY][ILV][CDEHKNQRST][ACDGNPSTV][ACFGHIKLMRTVWY][DEHKR][CDEHKNQRST][ACDGNPSTV]\w{0,6}[ACDGNPSTV]{0,2}[ACFGHIKLMRTVWY]{0,2}[CDEHKNQRST]\w[ACFGHIKLMRTVWY]{0,2}[ACDEGHKNQRST][CDEHKNQRST]L[ACDGNPSTV]\w[ILV]{0,2}\w[ILV]{0,2}[DEHKR]
DUF3452	\w{0,2}[ACFGHIKLMRTVWY]\w{0,15}[ACDEGHKNQRST]\w{0,12}[ACDGNPSTV]\w{0,2}[ACFGHIKLMRTVWY][ST][ILV][ACDGNPSTV][CDEHKNQRST][ILV]L[CDEHKNQRST][ACDEGHKNQRST]\w[CDEHKNQRST][ILV]\w{0,15}[ACDGNPSTV][ILV]\w[CDEHKNQRST]\wF[ACFGHIKLMRTVWY][CDEHKNQRST]\w{0,8}[DEHKR][ACFGHIKLMRTVWY][ACDEGHKNQRST][CDEHKNQRST][ACFGHIKLMRTVWY]\w{0,2}[CDEHKNQRST][ACFGHIKLMRTVWY]{0,2}[CDEHKNQRST]\w{0,12}[CDEHKNQRST][ACFGHIKLMRTVWY][ACDEGHKNQRST]\w{0,8}[ACDEGHKNQRST][ACFGHIKLMRTVWY]\w[CDEHKNQRST]{0,2}[ILV][CDEHKNQRST]{0,3}[ACFGHIKLMRTVWY][ACDGNPSTV][ILV]\w[ACFGHIKLMRTVWY]{0,2}[ILV][ACFGHIKLMRTVWY][CDEHKNQRST]\w[HKR][FHWY][CDEHKNQRST]\w[ACFGHIKLMRTVWY][FHWY]\w[ACDEGHKNQRST][ILV]F\w[ACDEGHKNQRST]\w{0,30}[ACDEGHKNQRST]\w{0,2}[ACDGNPSTV]\w{0,3}[CDEHKNQRST][ILV][ACFGHIKLMRTVWY][CDEHKNQRST][ACFGHIKLMRTVWY][ACDGNPSTV]W[ACFGHIKLMRTVWY][ILV]F[ILV][ACFGHIKLMRTVWY]{0,2}[HKR][ACDGNPSTV]\w[CDEHKNQRST][ACFGHIKLMRTVWY]\w{0,5}[ACDEGHKNQRST][DE]L[ILV]\w{0,6}[ACDGNPSTV]{0,2}[ACFGHIKLMRTVWY][CDEHKNQRST][ILV]L[ILV][ACDGNPSTV]{0,2}[ACFGHIKLMRTVWY][ACDGNPSTV][ACFGHIKLMRTVWY]{0,4}[CDEHKNQRST]
DUF1713	\w[ACFGHIKLMRTVWY][ACDEGHKNQRST][ACDGNPSTV][ILV][ACFGHIKLMRTVWY][DEHKR][ACFGHIKLMRTVWY]R[HKR][ACFGHIKLMRTVWY]KM[CDEHKNQRST][HKR]HK[ACFGHIKLMRTVWY][HKR]K[ACFGHIKLMRTVWY]{0,2}[HKR][ACDEGHKNQRST][CDEHKNQRST][HKR][ACFGHIKLMRTVWY]\w{0,3}[ACFGHIKLMRTVWY][CDEHKNQRST]{0,2}\w[ACDEGHKNQRST][CDEHKNQRST]
DUF3635	\w{0,2}[ACFGHIKLMRTVWY]F[CDEHKNQRST]\w{0,2}[AGS][CDEHKNQRST][ACDGNPSTV]\w{0,7}[DEHKR]\w{0,7}Q[ACFGHIKLMRTVWY][DE][ACFGHIKLMRTVWY]\w{0,2}YR[ACFGHIKLMRTVWY]M[HKR][ACDEGHKNQRST]\w*[ACDEGHKNQRST]{0,2}[CDEHKNQRST]\w{0,2}W[ACDEGHKNQRST]{0,2}[FHWY][ACDEGHKNQRST]P[ACFGHIKLMRTVWY]TN[ILV][ACFGHIKLMRTVWY]W[ILV]\wY[ILV][ACFG

	HIKLMRTVWY][CDEHKNQRST][ACFGHIKLMRTVWY]{0,2}\w[ACFGHIKLMRTVWY][CDEHKNQRST]\w{0,3}[CDEHKNQRST][ACFGHIKLMRTVWY][CDEHKNQRST]\w{0,9}[ACDEGHKNQRST]\w[ACFGHIKLMRTVWY][CDEHKNQRST][ACDEGHKNQRST][ACFGHIKLMRTVWY][CDEHKNQRST][ACDEGHKNQRST][ACFGHIKLMRTVWY]{0,2}[CDEHKNQRST]\w[ACFGHIKLMRTVWY]\w{0,9}[ACFGHIKLMRTVWY][CDEHKNQRST][ACDEGHKNQRST]\w{0,2}[ACDEGHKNQRST]\w[CDEHKNQRST]\w{0,3}[ACFGHIKLMRTVWY][ACDEGHKNQRST][CDEHKNQRST]\w[ACFGHIKLMRTVWY]{0,2}
DUF3454	[ACFGHIKLMRTVWY]\w[CDEHKNQRST][FHWY]\w[ST]PP[ST][CDEHKNQRST]H[ST]\w[ACDGNPSTV]\w*[ACDGNPSTV]P[CDEHKNQRST]\w[HKR]\w[ACFGHIKLMRTVWY][CDEHKNQRST][ACFGHIKLMRTVWY][ACDGNPSTV][CDEHKNQRST]\w*[ACDGNPSTV][FHWY]\wTPSPESP[ACDEGHKNQRST][CDEHKNQRST]WSS[ACDGNPSTV]SP[CDEHKNQRST]S\w{0,2}SDW\w{0,2}S[DE][ACDGNPSTV][ACFGHIKLMRTVWY][ACDGNPSTV]SP[ACDGNPSTV]{0,2}
DUF1716	\w[CDEHKNQRST][ACDEGHKNQRST][CDEHKNQRST]\w{0,2}[CDEHKNQRST][ACFGHIKLMRTVWY]\w[ACDEGHKNQRST][ACFGHIKLMRTVWY]\w[CDEHKNQRST][ACDEGHKNQRST]{0,2}\w*[ILV][ACDGNPSTV]\w[ACDEGHKNQRST]\w[ILV][HKR]{0,2}[ACFGHIKLMRTVWY]{0,2}[ILV][ACDEGHKNQRST][ACFGHIKLMRTVWY][DE][HKR][CDEHKNQRST][ACFGHIKLMRTVWY]\w[DEHKR]N[ACDEGHKNQRST][DEHKR]\wR[ACFGHIKLMRTVWY][HKR][FHWY][ACDGNPSTV]{0,2}[CDEHKNQRST]P[CDEHKNQRST]K\w{0,2}F[ACFGHIKLMRTVWY][CDEHKNQRST]SE[ACFGHIKLMRTVWY][DE]L[CDEHKNQRST][ACDEGHKNQRST]\w[ILV][CDEHKNQRST][ACDEGHKNQRST][ACFGHIKLMRTVWY][CDEHKNQRST]\w[ILV][AGS][ACDEGHKNQRST]\wP[CDEHKNQRST]\w{0,5}[ACFGHIKLMRTVWY]Y\w{0,2}[ACFGHIKLMRTVWY]{0,2}[CDEHKNQRST]\w[ACFGHIKLMRTVWY][ACDEGHKNQRST][ACDGNPSTV][ACFGHIKLMRTVWY]\w[CDEHKNQRST][ILV][ACFGHIKLMRTVWY][ACDEGHKNQRST]LL[ACDGNPSTV]H[CDEHKNQRST]N[ACDGNPSTV]D\w[ILV][ACDGNPSTV][ACFGHIKLMRTVWY][ACDGNPSTV]{0,2}[ILV]
DUF663	E[ACFGHIKLMRTVWY][DE]\w{0,7}[CDEHKNQRST]\w{0,2}[ACDEGHKNQRST]\w{0,7}[CDEHKNQRST]{0,3}[ACFGHIKLMRTVWY][ACDEGHKNQRST][CDEHKNQRST][ACFGHIKLMRTVWY]\w[DEHKR][ACDEGHKNQRST][ILV][HKR][CDEHKNQRST][ACFGHIKLMRTVWY]{0,2}[ACDGNPSTV]{0,2}\w[FHWY][DEHKR]\w{0,22}[CDEHKNQRST]\w{0,3}[CDEHKNQRST][FHWY][ACFGHIKLMRTVWY][DEHKR][ACFGHIKLMRTVWY]{0,2}[CDEHKNQRST]\w{0,6}[ACDEGHKNQRST][CDEHKNQRST][ACFGHIKLMRTVWY][CDEHKNQRST][ACDEGHKNQRST][ACDGNPSTV][DEHKR]\w[CDEHKNQRST]\w[ACFGHIKLMRTVWY]\w{0,33}[CDEHKNQRST]{0,2}\w{0,11}[ACDEGHKNQRST][ACDGNPSTV][ACFGHIKLMRTVWY]\w[ACDGNPSTV]\w{0,6}G\w[ACFGHIKLMRTVWY][ILV][CDEHKNQRST][ILV]\w{0,3}[ILV][ACDEGHKNQRST]{0,2}[ACFGHIKLMRTVWY]P\w[ACDEGHKNQRST][ACFGHIKLMRTVWY]{0,2}[CDEHKNQRST]{0,2}\w{0,24}[ACDEGHKNQRST]\w[ACDGNPSTV][ACFGHIKLMRTVWY]{0,2}[ILV][ACFGHIKLMRTVWY][AGS]L[ACFGHIKLMRTVWY]\w[ACDEGHKNQRST]E[CDEHKNQRST]{0,2}[ACFGHIKLMRTVWY][AGS][ACFGHIKLMRTVWY]{0,2}[CDEHKNQRST][ACFGHIKLMRTVWY]\w[ILV][CDEHKNQRST]{0,4}\w[ACFGHIKLMRTVWY][ACDEGHKNQRST]\w{0,4}[ACDEGHKNQRST]\w[ILV][CDEHKNQRST][ACDGNPSTV][CDEHKNQRST][DE]\w{0,3}[ILV]{0,2}[ACFGHIKLMRTVWY][CDEHKNQRST][ACFGHIKLMRTVWY]\w{0,2}G[ACFGHIKLMRTVWY]R{0,2}[FHWY]\w[ACFGHIKLMRTVWY]\wP[ILV][FHWY][AGS]\w[ACDEGHKNQRST]\w{0,9}[ACDGNPSTV]\w[ACDGNPSTV]{0,2}[ACFGHIKLMRTVWY][CDEHKNQRST][HKR][ACFGHIKLMRTVWY]\w[HKR][FHWY][ACFGHIKLMRTVWY]\w{0,2}[ACDEGHKNQRST]\w{0,5}[ACFGHIKLMRTVWY][ACDGNPSTV][ACFGHIKLMRTVWY][AGS][ST][ACFGHIKLMRTVWY]{0,2}[AGS]P[ILV]\w{0,2}[ACDGNPSTV]{0,2}\w{0,2}[ACDGNPSTV]{0,2}[ACFGHIKLMRTVWY][ILV][ACFGHIKLMRTVWY]{0,2}[CDEHKNQRST]\w{0,24}[ACDEGHKNQRST]\w{0,3}[ACDGNPSTV][ACFGHIKLMRTVWY][CDEHKNQRS

	T][ILV][ACFGHIKLMRTVWY][AGS][CDEHKNQRST]G[ACDGNPSTV][ACFGHIKLMRTVWY]{0,2}[CDEHKNQRST][ACFGHIKLMRTVWY][ACDGNPSTV]\w{0,2}[ACDGNPSTV][ACDEGHKNQRST]\w[ILV][ACFGHIKLMRTVWY]K[HKR][ILV][ACFGHIKLMRTVWY]L[ACDGNPSTV]G\w{0,10}[ACFGHIKLMRTVWY]P[ACFGHIKLMRTVWY][HKR][ILV]\wK[CDEHKNQRST][ACFGHIKLMRTVWY][ACDGNPSTV][ACFGHIKLMRTVWY][ILV][HKR]\wMF\w[ACDGNPSTV]\w{0,35}[DE][ILV][ACDEGHKNQRST][ACFGHIKLMRTVWY]F[DEHKR][ACDGNPSTV][ACFGHIKLMRTVWY]\w[ILV][ACFGHIKLMRTVWY]T[ACFGHIKLMRTVWY]\w{0,2}G[ACFGHIKLMRTVWY][CDEHKNQRST]G\w[ILV]K[DEHKR][ACDGNPSTV][ILV]\w{0,14}[ACDGNPSTV]{0,2}[DEHKR]G[ACFGHIKLMRTVWY]{0,2}[HKR][ACDGNPSTV][ACFGHIKLMRTVWY]F[DE][ACDGNPSTV][CDEHKNQRST][ILV]\w{0,2}[CDEHKNQRST]D[ACFGHIKLMRTVWY]V[ACFGHIKLMRTVWY]{0,2}[CDEHKNQRST][ACFGHIKLMRTVWY][FHWY][ACFGHIKLMRTVWY][CDEHKNQRST][ILV]
DUF1220	\w[CDEHKNQRST][ACDEGHKNQRST][CDEHKNQRST][ACDEGHKNQRST][ACDGNPSTV]\w[ACDGNPSTV]\w[CDEHKNQRST]\w{0,13}[ACDEGHKNQRST]\w{0,2}[ACDEGHKNQRST]\w[CDEHKNQRST][DEHKR]\w[DE][ILV]\w[CDEHKNQRST][DE]\w{0,6}S[ACFGHIKLMRTVWY][DEHKR][ACDEGHKNQRST][CDEHKNQRST]\w{0,2}T[ACDGNPSTV]{0,2}\w{0,3}[ACDEGHKNQRST]\w[ACDGNPSTV]{0,3}\w[CDEHKNQRST][ACDGNPSTV][ACFGHIKLMRTVWY][ACDEGHKNQRST][ACDGNPSTV]\w[ACDGNPSTV]\w[ACFGHIKLMRTVWY]{0,2}[ACDGNPSTV]\w[DE]\w{0,3}[DEHKR][CDEHKNQRST]{0,2}[ACFGHIKLMRTVWY][ACDGNPSTV]\w[ACDGNPSTV][ACFGHIKLMRTVWY]\w[ACFGHIKLMRTVWY][ACDGNPSTV]
Zinc finger CCHC type	\wC[ACFGHIKLMRTVWY][ACDEGHKNQRST]C[ACDGNPSTV][CDEHKNQRST]\w{0,2}GH[ACFGHIKLMRTVWY][ACDEGHKNQRST][CDEHKNQRST]{0,2}C\w[CDEHKNQRST]
RAB5 / RAB5	[ACFGHIKLMRTVWY]K[ILV]{0,4}G[DE][ACDGNPSTV][AGS]VGK[ST][ACDGNPSTV][ILV][ACFGHIKLMRTVWY]\w[CDEHKNQRST][FHWY][ACDGNPSTV][CDEHKNQRST][ACDGNPSTV][CDEHKNQRST]F\w{0,2}[CDEHKNQRST]\w{0,3}[ACDGNPSTV]T[ILV]G[ILV]F[ACDEGHKNQRST]\w[HKR]\w[ILV][CDEHKNQRST][ACFGHIKLMRTVWY]\w{0,10}[CDEHKNQRST][ACDGNPSTV][CDEHKNQRST][ACFGHIKLMRTVWY][ILV][HKR][ACFGHIKLMRTVWY]Q[ILV]WDTAGQER[FHWY][CDEHKNQRST]\w[ACDGNPSTV][ILV][ACDGNPSTV][ACDEGHKNQRST][AGS]YYR[ACDGNPSTV]A\w[AGS][ACFGHIKLMRTVWY][ILV]{0,3}YD[ILV][ST][CDEHKNQRST][ACDEGHKNQRST]{0,2}[ST]F[CDEHKNQRST]{0,2}[ILV][ACDEGHKNQRST][CDEHKNQRST]\wW[ACFGHIKLMRTVWY][CDEHKNQRST][DE][ACFGHIKLMRTVWY][CDEHKNQRST]\w[ACFGHIKLMRTVWY][ACDGNPSTV]{0,2}\w{0,5}[ACDGNPSTV][ACFGHIKLMRTVWY]\w[ACFGHIKLMRTVWY]{0,2}L[ILV]GNK\wD[ACFGHIKLMRTVWY][ACDEGHKNQRST][CDEHKNQRST]\w{0,3}[HKR][ACDEGHKNQRST]V[ACDGNPSTV]\w[CDEHKNQRST]{0,2}[AGS][ACDEGHKNQRST]{0,2}[ACFGHIKLMRTVWY]\w{0,13}[AGS][CDEHKNQRST]{0,2}\w[ACDEGHKNQRST][ACFGHIKLMRTVWY]\w[FHWY][ACFGHIKLMRTVWY]\wE[ACDGNPSTV]SA[ACFGHIKLMRTVWY][CDEHKNQRST][ACDGNPSTV]\w[ACDGNPSTV]V[CDEHKNQRST]{0,2}\w{0,3}[ACDGNPSTV]F\w[ACDEGHKNQRST][ILV][ACFGHIKLMRTVWY][CDEHKNQRST]{0,2}[ILV][ACFGHIKLMRTVWY][ACDEGHKNQRST]\w
VPS9	\w[ACFGHIKLMRTVWY][DEHKR][ILV][ACDEGHKNQRST][CDEHKNQRST][ACFGHIKLMRTVWY][DEHKR]\w[CDEHKNQRST][ACFGHIKLMRTVWY]{0,2}[CDEHKNQRST][ACFGHIKLMRTVWY]\w[ACFGHIKLMRTVWY]\w{0,2}SP[CDEHKNQRST][ACDEGHKNQRST][CDEHKNQRST][ILV][ACFGHIKLMRTVWY]{0,2}[ILV]L[CDEHKNQRST][ACDGNPSTV]{0,2}[DEHKR][ILV]{0,2}[ACFGHIKLMRTVWY]{0,4}[DEHKR]\w[CDEHKNQRST][ACDEGHKNQRST]\w{0,2}[ACDEGHKNQRST]\w{0,2}[AGS]AD[ACDGNPSTV][FHWY][ILV]P[ILV]L\w[ACFGHIKLMRTVWY][ACDGNPSTV][ILV][ACFGHIKLMRTVWY][CDEHKNQRST][ACDGNPSTV]{0,2}\w{0,2}[ACDEGHKNQRST]L[ACFGHIKLMRTVWY]\w[CD

	EHKNQRST][ACDGNPSTV][CDEHKNQRST][FHWY][ACFGHIKLMRTVWY][CDEHKNQRST]{0,2}[ACFGHIKLMRTVWY]{0,2}[ACDEGHKNQRST][ACDGNPSTV][CDEHKNQRST][ACFGHIKLMRTVWY][ILV][CDEHKNQRST]G[DEHKR][ACDEGHKNQRST][AGS]YYL[ST][ACDGNPSTV][ACFGHIKLMRTVWY][CDEHKNQRST][AGS]{0,2}L[ACDGNPSTV][ACFGHIKLMRTVWY][ILV][CDEHKNQRST][ACDGNPSTV][ILV][ACDEGHKNQRST][CDEHKNQRST][ACDEGHKNQRST][CDEHKNQRST]\w{0,11}
Allergen V5/ TpX1 family signature	[ACDEGHKNQRST]\w{0,2}[CDEHKNQRST][ACDEGHKNQRST][ACFGHIKLMRTVWY][ILV][CDEHKNQRST]\w{0,2}HN[ACDEGHKNQRST][ACFGHIKLMRTVWY]R[ACDEGHKNQRST]{0,2}[ILV][ACDGNPSTV]\w{0,30}[ACFGHIKLMRTVWY]\w{0,2}[ACFGHIKLMRTVWY]\w{0,2}W[ACDGNPSTV][ACDEGHKNQRST][CDEHKNQRST][ILV][ACDEGHKNQRST]{0,2}\wA[CDEHKNQRST][ACDEGHKNQRST][FHWY]A[CDEHKNQRST]{0,2}\w{0,6}C\w[ACFGHIKLMRTVWY]\w{0,3}H[ACDGNPSTV]\w{0,15}[ACFGHIKLMRTVWY]G[DEHKR]N[ILV][ACFGHIKLMRTVWY]{0,3}\w{0,13}[ACFGHIKLMRTVWY][ACDEGHKNQRST]\w[AGS][ACFGHIKLMRTVWY][ACDEGHKNQRST]\wW\w[CDEHKNQRST]E[ACFGHIKLMRTVWY][ACDEGHKNQRST]{0,2}[ACFGHIKLMRTVWY][ACDGNPSTV][ACFGHIKLMRTVWY][ACDEGHKNQRST]{0,2}\w{0,14}[ACFGHIKLMRTVWY]{0,2}[AGS]H[ACFGHIKLMRTVWY]TQ[ACFGHIKLMRTVWY][ACDGNPSTV]W[ACDEGHKNQRST][CDEHKNQRST][ST]\w[ACDEGHKNQRST][ILV]GC[AGS]\w[ACFGHIKLMRTVWY]\w{0,2}[ACDGNPSTV]\w[ACDEGHKNQRST]{0,2}\w{0,16}[ACFGHIKLMRTVWY]{0,3}[ACDGNPSTV]C[CDEHKNQRST]Y[ACDEGHKNQRST][ACDGNPSTV]\wGN[ACFGHIKLMRTVWY]\w[ACDEGHKNQRST]
Integrin	[ACFGHIKLMRTVWY]{0,3}\w{0,2}[ACFGHIKLMRTVWY][DEHKR][CDEHKNQRST][ACDGNPSTV]\w{0,4}[ACDGNPSTV]\w[ACFGHIKLMRTVWY]\w[ACFGHIKLMRTVWY][CDEHKNQRST]\w[ACDGNPSTV]\w[ACFGHIKLMRTVWY][CDEHKNQRST][ACDGNPSTV]\wE[ACDGNPSTV]{0,2}G\w{0,2}[ACFGHIKLMRTVWY]\w[ACFGHIKLMRTVWY]\w[ILV][ACFGHIKLMRTVWY][HKR][CDEHKNQRST][ACDGNPSTV]{0,2}\w{0,3}[ACFGHIKLMRTVWY][CDEHKNQRST][ACDEGHKNQRST]\w[ACDGNPSTV]\w[ILV][ACDGNPSTV][ACFGHIKLMRTVWY]\wT\w[ACDGNPSTV]{0,2}[ST]A[CDEHKNQRST][ACDEGHKNQRST][ACDGNPSTV]\w[ACDEGHKNQRST]D[FHWY]\w{0,2}[ACFGHIKLMRTVWY]\w{0,3}[ACDGNPSTV][CDEHKNQRST][ILV]\w[FHWY]\w[ACDGNPSTV]{0,2}[CDEHKNQRST]\w[ACDGNPSTV]\w[CDEHKNQRST]{0,2}[ILV][CDEHKNQRST][ILV]\w[ILV]{0,2}\w{0,4}[ACDGNPSTV]D[CDEHKNQRST]\w[ACFGHIKLMRTVWY][DEHKR][ACDEGHKNQRST]\w{0,2}[ACDGNPSTV][DE][CDEHKNQRST]F[ACFGHIKLMRTVWY][ILV]\w[ILV][ACDEGHKNQRST]
AroQ CM	R[ACDEGHKNQRST]{0,2}[ILV][ACDGNPSTV][ACDEGHKNQRST][ILV]D[ACDEGHKNQRST][CDEHKNQRST][ILV][ACFGHIKLMRTVWY][ACDEGHKNQRST][ILV]{0,2}[ACDEGHKNQRST][CDEHKNQRST]R[ACFGHIKLMRTVWY][ACDEGHKNQRST][ACFGHIKLMRTVWY][ACDGNPSTV][ACDEGHKNQRST][CDEHKNQRST][ILV][AGS][ACDEGHKNQRST][ACFGHIKLMRTVWY]K\w[ACDEGHKNQRST]\w*[ACDEGHKNQRST]\w{0,4}[ACFGHIKLMRTVWY]\w[ACDEGHKNQRST]\w[ACDEGHKNQRST]R[ACDEGHKNQRST]{0,3}[ILV][ACFGHIKLMRTVWY][ACDEGHKNQRST][CDEHKNQRST][ACFGHIKLMRTVWY]{0,2}[ACDEGHKNQRST]\w[ACDGNPSTV]\w{0,4}[ACDGNPSTV]\w{0,6}[ACFGHIKLMRTVWY]\w{0,2}[ACDEGHKNQRST]\w[ACFGHIKLMRTVWY][ACDEGHKNQRST]{0,2}[ACFGHIKLMRTVWY]{0,2}[CDEHKNQRST]\w[ACFGHIKLMRTVWY]{0,2}[CDEHKNQRST]\w[ACDGNPSTV][ACFGHIKLMRTVWY]\w[ACFGHIKLMRTVWY][CDEHKNQRST]\w{0,3}
PAN domain	\w{0,4}[ACDEGHKNQRST]\w[FHWY]\w[ACFGHIKLMRTVWY]{0,2}\w[ACDEGHKNQRST][ACFGHIKLMRTVWY]\w[ACFGHIKLMRTVWY]\w*[ACDGNPSTV]\w[CDEHKNQRST]{0,2}C[ACFGHIKLMRTVWY][ACDEGHKNQRST][ACFGHIKLMRTVWY]C\w[CDEHKNQRST]\w*[ACDEGHKNQRST]\w{0,2}C[CDEHKNQRST][AGS][ACFGHIKLMRTVWY]\w[FHWY]\w*[ACDEGHKNQRST]{0,3}C\w[ACFGHIKLMRTVWY]\w[ACFGHIKLMRTVWY]\w{0,3}[ACFGHIKLMRTVWY]\w{0,3}[ACFGHIKLMRTVWY]\w*[ACFGHIKLMRTVWY]\w[ACFGHIKLMRTVWY]\w{0,2}[ACFGHIKLMRTVWY]{0,2}\w[CDEHKNQRST]\w

MD2	[ACFGHIKLMRTVWY][CDEHKNQRST]\wC[ACDGNPSTV]\w*[ACFGHIKLMRTVWY]\w[ACFGHIKLMRTVWY]\w[ACDEGHKNQRST][ILV][CDEHKNQRST][ILV][CDEHKNQRST][ACDGNPSTV]{0,3}\w*[ACFGHIKLMRTVWY][ACDGNPSTV][CDEHKNQRST]\w[ACFGHIKLMRTVWY][CDEHKNQRST][ACFGHIKLMRTVWY][CDEHKNQRST][ACFGHIKLMRTVWY][ACDEGHKNQRST]\w{0,2}[ACFGHIKLMRTVWY]\w{0,2}[ACDEGHKNQRST][CDEHKNQRST]{0,2}[ACFGHIKLMRTVWY][ACDEGHKNQRST]{0,2}[ACFGHIKLMRTVWY]\w{0,2}[ACFGHIKLMRTVWY]{0,2}\w[ILV][ACDEGHKNQRST][ACFGHIKLMRTVWY]\w[ACFGHIKLMRTVWY]\w*[ILV]\w[ACFGHIKLMRTVWY]\w*[ACDGNPSTV][ACFGHIKLMRTVWY]C[CDEHKNQRST]\w*C[ACDGNPSTV]\w{0,4}[ACFGHIKLMRTVWY]\w{0,2}G[CDEHKNQRST]\w{0,3}[ACFGHIKLMRTVWY]\w{0,10}[ACFGHIKLMRTVWY]\w{0,3}[ACFGHIKLMRTVWY]P\w[ACFGHIKLMRTVWY]\w{0,6}[CDEHKNQRST][ACFGHIKLMRTVWY]\w[ACFGHIKLMRTVWY]\w[ACFGHIKLMRTVWY][ACDEGHKNQRST][ACFGHIKLMRTVWY]{0,2}[ACDGNPSTV]\w{0,7}[CDEHKNQRST][ACDEGHKNQRST]{0,2}\w[ACFGHIKLMRTVWY]{0,2}C[ACFGHIKLMRTVWY]\w[ACFGHIKLMRTVWY]\w[ACFGHIKLMRTVWY]\w{0,3}
RNAse Pc	[ACDEGHKNQRST]\w[ACDGNPSTV]\w[ACFGHIKLMRTVWY]\w[ACFGHIKLMRTVWY]F\w[ACFGHIKLMRTVWY]QH[ACFGHIKLMRTVWY][ACDGNPSTV]{0,2}[ACDEGHKNQRST]\w{0,10}[ACFGHIKLMRTVWY]C[ACDGNPSTV]\w[ACFGHIKLMRTVWY]M[CDEHKNQRST][ACDEGHKNQRST]{0,2}[CDEHKNQRST][ACFGHIKLMRTVWY]{0,2}[CDEHKNQRST][ACDEGHKNQRST]\w[CDEHKNQRST]CK[ACDGNPSTV][ACFGHIKLMRTVWY]NTF[ILV]H[CDEHKNQRST][ACDGNPSTV][ACFGHIKLMRTVWY][ACDGNPSTV]{0,2}[ILV][ACDEGHKNQRST][ACDGNPSTV][ILV]C\w[CDEHKNQRST][ACDEGHKNQRST][CDEHKNQRST]\w{0,3}[ACFGHIKLMRTVWY]\wC\w{0,5}[ACDEGHKNQRST][CDEHKNQRST]NC[FHWY][CDEHKNQRST]S\w[ACDGNPSTV][ACDEGHKNQRST][ACFGHIKLMRTVWY][CDEHKNQRST][ILV]T[CDEHKNQRST]C[CDEHKNQRST]\w[CDEHKNQRST][AGS][ACDGNPSTV]\w{0,2}[CDEHKNQRST]{0,2}[ACFGHIKLMRTVWY][ACDGNPSTV]{0,2}C[ACDEGHKNQRST]Y[CDEHKNQRST][ACDGNPSTV]{0,2}[ACDEGHKNQRST]{0,2}[CDEHKNQRST][HKR][ACFGHIKLMRTVWY]{0,2}[ILV]VAC[DE][ACDGNPSTV][ACDEGHKNQRST]\w{0,11}[ILV]PVH[ACFGHIKLMRTVWY]D[ACDEGHKNQRST]\w[ACFGHIKLMRTVWY]\w{0,2}
LRR RI	[ACDGNPSTV]\w[ACDEGHKNQRST]L[CDEHKNQRST]{0,2}[ILV][ACDGNPSTV][ILV][ACDEGHKNQRST]{0,2}C\w{0,3}[ILV][ST][DEHKR]\w{0,4}[AGS][ILV]\w[ACDEGHKNQRST][ILV][ACDGNPSTV][CDEHKNQRST]\w
G alpha	\w[ACDEGHKNQRST][ACFGHIKLMRTVWY][ACDGNPSTV][CDEHKNQRST][ACDEGHKNQRST][ILV][CDEHKNQRST]{0,2}\w[ILV][CDEHKNQRST][ACDEGHKNQRST][CDEHKNQRST]{0,3}[ACDEGHKNQRST]\w[ACDEGHKNQRST]\w{0,22}[ACFGHIKLMRTVWY]K[ILV]L{0,3}G[ACDGNPSTV][AGS]ESGKST[ILV][ACFGHIKLMRTVWY]KQ[ACFGHIKLMRTVWY][HKR][ILV]{0,2}[FHWY]\w[ACDEGHKNQRST][ACDGNPSTV]\w[FHWY][ACDGNPSTV][ACDEGHKNQRST][CDEHKNQRST]\w[DE][ACFGHIKLMRTVWY]\w[ACDEGHKNQRST][ACFGHIKLMRTVWY][ACDEGHKNQRST]\w[ACFGHIKLMRTVWY][ILV][ACFGHIKLMRTVWY][ACDEGHKNQRST]N[ACFGHIKLMRTVWY]{0,2}[CDEHKNQRST][ACDGNPSTV][ACFGHIKLMRTVWY][ACDEGHKNQRST]\w[ILV]{0,2}[CDEHKNQRST][ACDGNPSTV][ACFGHIKLMRTVWY][ACDEGHKNQRST]\w[ACDEGHKNQRST][ACFGHIKLMRTVWY][ACDEGHKNQRST][ACFGHIKLMRTVWY]\w[ACFGHIKLMRTVWY][ACDEGHKNQRST]\w{0,16}[CDEHKNQRST][ACFGHIKLMRTVWY][CDEHKNQRST][ACFGHIKLMRTVWY]{0,3}[ACDEGHKNQRST]\w{0,8}[ACDEGHKNQRST]\w{0,2}[ACDEGHKNQRST]\w*[ACFGHIKLMRTVWY]\w{0,2}[ACDEGHKNQRST][ACFGHIKLMRTVWY]{0,2}[ACDEGHKNQRST]\w[ACFGHIKLMRTVWY][CDEHKNQRST][ACDEGHKNQRST]LW[ACDEGHKNQRST]\w[ACDGNPSTV]\w[ACDGNPSTV][ILV][CDEHKNQRST][ACDEGHKNQRST][ACFGHIKLMRTVWY]{0,2}[ACDEGHKNQRST]\w{0,2}[DEHKR][ACDEGHKNQRST][CDEHKNQRST]{0,2}[FHWY]\w{0,3}[ACDEGHKNQRST][ILV]\wD[ACDGNPSTV]\w{0,28}[ACFGHIKLMRTVWY]\wY[FHWY][ACFGHIKLMRTVWY][ACDGNPSTV][CDEHKNQR

	ST][ILV][ACDEGHKNQRST]R[ILV][ACDEGHKNQRST][CDEHKNQRST]\w[CDEHKNQRST]Y[ACFGHIKLMRTVWY]P[ST][ACDEGHKNQRST][CDEHKNQRST]D[ILV]L[ACFGHIKLMRTVWY][ACDGNPSTV]R[ACFGHIKLMRTVWY]\wT[CDEHKNQRST]G[ILV]\w[DE][ACFGHIKLMRTVWY]\wF\w[ACFGHIKLMRTVWY][CDEHKNQRST][ACDEGHKNQRST]\w{0,2}[ACFGHIKLMRTVWY]\w{0,19}[DE][ACFGHIKLMRTVWY]{0,2}DVG{0,2}QR[ACDGNPSTV][CDEHKNQRST]R[HKR]KW[ILV][CDEHKNQRST]\w{0,4}[ACFGHIKLMRTVWY]F[DE][ACDGNPSTV]\w[ILV][ACDGNPSTV][AGS][ILV]{0,2}F[ACFGHIKLMRTVWY][ACDGNPSTV][AGS][ACFGHIKLMRTVWY][ST][ACDEGHKNQRST]YD[CDEHKNQRST][ACFGHIKLMRTVWY]L\wE[DE][ACDEGHKNQRST][CDEHKNQRST]\w*NR[ACFGHIKLMRTVWY][ACDEGHKNQRST]E[AGS][ACFGHIKLMRTVWY]\w[ILV]F[CDEHKNQRST]{0,2}[ILV][ACFGHIKLMRTVWY][ACDGNPSTV]{0,2}\w[ACFGHIKLMRTVWY]F\w[CDEHKNQRST]\w[ACDGNPSTV]{0,2}[ACFGHIKLMRTVWY][ILV]LFLNK[ACFGHIKLMRTVWY]D[ILV][ACFGHIKLMRTVWY][ACDEGHKNQRST][DEHKR]K[ILV]\w[ACDEGHKNQRST][ACDGNPSTV]\w{0,4}[ACFGHIKLMRTVWY][CDEHKNQRST]\w[ACFGHIKLMRTVWY]F[ACDGNPSTV][CDEHKNQRST][FHWY][CDEHKNQRST]G\w{0,16}[ACDEGHKNQRST][ACDGNPSTV][ACFGHIKLMRTVWY][CDEHKNQRST][ACDEGHKNQRST][ACDGNPSTV]\w{0,2}[FHWY][ACFGHIKLMRTVWY]\w[ACDEGHKNQRST]\wF\w[CDEHKNQRST][ACFGHIKLMRTVWY][ACDEGHKNQRST]\w{0,18}[HKR]\w[ACFGHIKLMRTVWY]Y\wH[ACFGHIKLMRTVWY]T[ACDGNPSTV]\wA[ACDGNPSTV]DT[CDEHKNQRST]{0,2}[ILV][CDEHKNQRST][ACFGHIKLMRTVWY][ILV][ACFGHIKLMRTVWY][ACDEGHKNQRST][ACDGNPSTV]{0,2}[ACFGHIKLMRTVWY][DE][ACFGHIKLMRTVWY][ILV][ACFGHIKLMRTVWY][CDEHKNQRST]{0,2}[ACDGNPSTV][ILV][CDEHKNQRST][ACDEGHKNQRST][ACFGHIKLMRTVWY]{0,3}
ANX	GT[ACDGNPSTV][DE][ACDEGHKNQRST][ACFGHIKLMRTVWY][ILV][ACFGHIKLMRTVWY][CDEHKNQRST][ILV][ACFGHIKLMRTVWY][ACDGNPSTV][CDEHKNQRST]R[ACDGNPSTV][ACDEGHKNQRST]\w[CDEHKNQRST][ACFGHIKLMRTVWY]\w[ACDEGHKNQRST][ILV][ACDEGHKNQRST]{0,3}[FHWY][ACDEGHKNQRST][CDEHKNQRST]\w[ACFGHIKLMRTVWY][ACDEGHKNQRST][CDEHKNQRST][ACDGNPSTV]\wL\w[CDEHKNQRST][ACDEGHKNQRST][ILV][ACDEGHKNQRST]{0,2}[DE][ACFGHIKLMRTVWY][ACDGNPSTV]G[CDEHKNQRST][ACFGHIKLMRTVWY][DEHKR]\w[DEHKR][ACFGHIKLMRTVWY][ILV]{0,2}[ACFGHIKLMRTVWY][ILV]
FN3	\w{0,3}[ACDGNPSTV]\w[ACDEGHKNQRST]\w{0,3}[ACFGHIKLMRTVWY][ACDEGHKNQRST][ACFGHIKLMRTVWY]\w{0,3}[ACDEGHKNQRST]{0,3}\w{0,6}[ACDEGHKNQRST][ACFGHIKLMRTVWY]\w[ILV][CDEHKNQRST]W[CDEHKNQRST]\w{0,3}[ACDGNPSTV]\w*[ACFGHIKLMRTVWY]{0,2}[ACDEGHKNQRST][ACFGHIKLMRTVWY]\w*[ACFGHIKLMRTVWY]\w*[ACDEGHKNQRST][ACFGHIKLMRTVWY]\w[ACFGHIKLMRTVWY][ACDEGHKNQRST]{0,2}\w{0,2}L\w{0,2}[ACDEGHKNQRST]\w{0,4}[ACDEGHKNQRST]\wY\w[ACFGHIKLMRTVWY][CDEHKNQRST][ILV][ACFGHIKLMRTVWY][ACDGNPSTV][ACFGHIKLMRTVWY]\w{0,5}[ACDGNPSTV]\w[ACDGNPSTV]
BRCT	\w{0,3}[ACFGHIKLMRTVWY][ACDEGHKNQRST][ACDGNPSTV]\w{0,5}[ACFGHIKLMRTVWY]\w[ACFGHIKLMRTVWY]{0,3}\w*[ACDEGHKNQRST]\w{0,2}[ACFGHIKLMRTVWY][ACDEGHKNQRST]\w[ACFGHIKLMRTVWY]\w[ACDEGHKNQRST][ACFGHIKLMRTVWY]{0,2}\w{0,2}[ACFGHIKLMRTVWY][ACDGNPSTV][AGS][CDEHKNQRST][ACFGHIKLMRTVWY]\w{0,2}[ACDEGHKNQRST]\w*[ACDEGHKNQRST][ACFGHIKLMRTVWY][ACDGNPSTV][ACFGHIKLMRTVWY]{0,2}[ILV][ACFGHIKLMRTVWY]\w[CDEHKNQRST]\w*[ACFGHIKLMRTVWY]\w{0,3}[ACDEGHKNQRST][ACFGHIKLMRTVWY]\w[ACFGHIKLMRTVWY][ILV][CDEHKNQRST]\w{0,4}[CDEHKNQRST][FHWY][ACFGHIKLMRTVWY]{0,2}[ACDEGHKNQRST]\w[ACFGHIKLMRTVWY][ACDEGHKNQRST]{0,3}

<table>
<tr><td>TPR</td><td>\w{0,2}[ACFGHIKLMRTVWY]{0,2}\w[ACDEGHKNQRST][ACFGHIKLMRTVWY][AGS]\w[ACFGHIKLMRTVWY]{0,2}\w{0,3}[ACDEGHKNQRST][CDEHKNQRST][ACFGHIKLMRTVWY][CDEHKNQRST]{0,2}A[ACFGHIKLMRTVWY][ACDEGHKNQRST]\w[ACFGHIKLMRTVWY][CDEHKNQRST]{0,2}[ACDGNPSTV][ILV][ACDEGHKNQRST][ACFGHIKLMRTVWY][ACDEGHKNQRST]\w[CDEHKNQRST]\w</td></tr>
<tr><td>SEL1</td><td>[ACDGNPSTV]\w[AGS]\w[ACFGHIKLMRTVWY]\w[ILV][AGS]\w[ACFGHIKLMRTVWY][FHWY]\w{0,2}G\w*[ACFGHIKLMRTVWY]{0,2}\w{0,2}[ACDGNPSTV]\w[ACDEGHKNQRST]\wA[ACFGHIKLMRTVWY]\w[ACFGHIKLMRTVWY]{0,2}[CDEHKNQRST]\w[AGS][ACDGNPSTV][ACDEGHKNQRST]\w[ACDEGHKNQRST]\w</td></tr>
<tr><td>HAT</td><td>[ACDEGHKNQRST]{0,2}[ACFGHIKLMRTVWY][CDEHKNQRST|ACDEGHKNQRST|ACFGHIKLMRTVWY]{0,2}[FHWY][CDEHKNQRST]{0,2}[ACFGHIKLMRTVWY]{0,2}\w*[ACDEGHKNQRST][ACFGHIKLMRTVWY]W\w[ACDEGHKNQRST][FHWY][ACFGHIKLMRTVWY][ACDEGHKNQRST][ACFGHIKLMRTVWY]\w{0,2}[ACDEGHKNQRST]</td></tr>
<tr><td>CY</td><td>\w[ACFGHIKLMRTVWY]\w[AGS][ACDGNPSTV]\w*[ACDGNPSTV]{0,2}\w[CDEHKNQRST|ACFGHIKLMRTVWY]\w[CDEHKNQRST|ACFGHIKLMRTVWY][ACFGHIKLMRTVWY][CDEHKNQRST|ACFGHIKLMRTVWY][ACDGNPSTV][ACFGHIKLMRTVWY][ACDEGHKNQRST][CDEHKNQRST|ACFGHIKLMRTVWY]N[CDEHKNQRST]{0,3}\w{0,2}[ACDGNPSTV][ACDEGHKNQRST]\w[ACFGHIKLMRTVWY]\w{0,4}[ACFGHIKLMRTVWY][CDEHKNQRST][ACFGHIKLMRTVWY]{0,2}[CDEHKNQRST]\w{0,2}[CDEHKNQRST]{0,2}[ILV][ACDGNPSTV]{0,2}G[ACFGHIKLMRTVWY]\w[FHWY][ACFGHIKLMRTVWY]{0,2}\w[CDEHKNQRST][ACFGHIKLMRTVWY][ACDEGHKNQRST][ACFGHIKLMRTVWY][ACDEGHKNQRST]\w*[CDEHKNQRST][ACDGNPSTV][ACDEGHKNQRST][ACDGNPSTV]\w[ACDEGHKNQRST]\w*[ACDEGHKNQRST]{0,2}[CDEHKNQRST]\w*[ILV][ACFGHIKLMRTVWY]\w[ACFGHIKLMRTVWY]\w[ACFGHIKLMRTVWY]\w*[CDEHKNQRST]\w[ACFGHIKLMRTVWY][ACDEGHKNQRST]\w{0,2}[ACDEGHKNQRST]\w[CDEHKNQRST][ACFGHIKLMRTVWY]\w</td></tr>
<tr><td>HhH1</td><td>[ACDEGHKNQRST]{0,2}[ILV]\w[ACDEGHKNQRST][ILV]\wG[ILV]G\w[ACDEGHKNQRST][ACFGHIKLMRTVWY][ACDGNPSTV][ACDEGHKNQRST]{0,2}[ILV][ACFGHIKLMRTVWY][ACDEGHKNQRST]</td></tr>
</table>
